# Global landscape of SARS-CoV-2 genomic surveillance and data sharing

**DOI:** 10.1038/s41588-022-01033-y

**Published:** 2022-03-28

**Authors:** Zhiyuan Chen, Andrew S. Azman, Xinhua Chen, Junyi Zou, Yuyang Tian, Ruijia Sun, Xiangyanyu Xu, Yani Wu, Wanying Lu, Shijia Ge, Zeyao Zhao, Juan Yang, Daniel T. Leung, Daryl B. Domman, Hongjie Yu

**Affiliations:** 1 Department of Infectious Diseases, Huashan Hospital, School of Public Health, Fudan University, Shanghai, China; 2 Key Laboratory of Public Health Safety, Fudan University, Ministry of Education, Shanghai, China; 3 Department of Epidemiology, Johns Hopkins Bloomberg School of Public Health, Baltimore, MD USA; 4 Institute of Global Health, Faculty of Medicine, University of Geneva, Geneva, Switzerland; 5 School of Public Health, School of Medicine, Shanghai Jiao Tong University, Shanghai, China; 6 Division of Infectious Diseases, University of Utah School of Medicine, Salt Lake City, UT USA; 7 Division of Microbiology & Immunology, University of Utah School of Medicine, Salt Lake City, UT USA; 8 Center for Global Health, Department of Internal Medicine, University of New Mexico Health Sciences Center, Albuquerque, NM USA; 9 Shanghai Institute of Infectious Disease and Biosecurity, Fudan University, Shanghai, China; 10 National Medical Center for Infectious Diseases, Huashan Hospital, Fudan University, Shanghai, China

**Keywords:** Infectious diseases, Epidemiology

## Abstract

Genomic surveillance has shaped our understanding of severe acute respiratory syndrome coronavirus 2 (SARS-CoV-2) variants. We performed a global landscape analysis on SARS-CoV-2 genomic surveillance and genomic data using a collection of country-specific data. Here, we characterize increasing circulation of the Alpha variant in early 2021, subsequently replaced by the Delta variant around May 2021. SARS-CoV-2 genomic surveillance and sequencing availability varied markedly across countries, with 45 countries performing a high level of routine genomic surveillance and 96 countries with a high availability of SARS-CoV-2 sequencing. We also observed a marked heterogeneity of sequencing percentage, sequencing technologies, turnaround time and completeness of released metadata across regions and income groups. A total of 37% of countries with explicit reporting on variants shared less than half of their sequences of variants of concern (VOCs) in public repositories. Our findings indicate an urgent need to increase timely and full sharing of sequences, the standardization of metadata files and support for countries with limited sequencing and bioinformatics capacity.

## Abstract (web summary)

Analyses on the global diversity of SARS-CoV-2 genomic surveillance across 118 countries and the extent of public availability of genomic data provide evidence to better inform SARS-CoV-2 surveillance policy.

## Main

Following the first pandemic wave of coronavirus disease 2019 (COVID-19), the emergence and dissemination of SARS-CoV-2 variants have resulted in new waves of infections across the globe. Some SARS-CoV-2 variants disappeared immediately, whereas others characterized by several key mutations adapted well, enabling their rapid spread^1^. As of 10 January 2022, the World Health Organization (WHO) has designated five VOCs associated with increased transmissibility and various extents of immune escape^2–4^, namely the Alpha, Beta, Gamma, Delta and Omicron variants, first detected in the United Kingdom, South Africa, Brazil, India and multiple countries, respectively^5^. Specifically, the Delta variant is highly transmissible, with an estimated transmissibility increase of 50–80% compared with the Alpha variant^6,7^, whereas the Beta variant has been shown to have a high reduction in neutralization activity, whether from natural infection or vaccination^8^, both reflected in lower vaccine efficacy or effectiveness^9–11^. At the time of writing, the Omicron variant was found to harbor multiple concerning mutations (e.g., P681H, E484A and T478K), which might be associated with a higher transmissibility and reinfection risk in comparison to other VOCs^12,13^.

The identification and classification of SARS-CoV-2 variants mainly relied on partial or whole-genome sequencing, although polymerase chain reaction (PCR) assays have been used to identify specific features relatively unique in specific variants, like S-gene target failure (SGTF)^14^ in the Alpha and Omicron variant. Since the first SARS-CoV-2 sequence was published in January 2020 (ref. ^15^), the unprecedented rate of genome data generation was far greater than any other pathogen^16^, with 4.8 million genomes deposited in Global Initiative on Sharing All Influenza Data (GISAID) through 31 October 2021 (ref. ^17^). Genomic surveillance has been vital to the early detection of mutations, monitoring of virus evolution and evaluating the degree of similarities between circulating variants with vaccine strains, especially since SARS-CoV-2 vaccines became available^18^.

Several studies have used genomic data to examine the evolution and associated spread of dominant variants in specific countries or regions, raising a claim of the rapidity of local transmission of SARS-CoV-2 variants and urgency of genomic surveillance^6,16,19–21^. However, the paucity of genomic data from low- and middle-income countries in these studies was concerning^6,19,20^. The impact of genome data is dependent on their quality, and the reliability and accuracy of such data may influence the global community’s ability to track the emergence and spread of variants in a timely manner.

In this study, we aimed to investigate the global diversity of SARS-CoV-2 genomic surveillance, the global distribution, properties and extent of public availability of genomes. In addition, we sought to map the global identification and spread of SARS-CoV-2 variants. These data can provide evidence to better inform SARS-CoV-2 surveillance policy.

## Results

**Extended Data Fig. 1:**
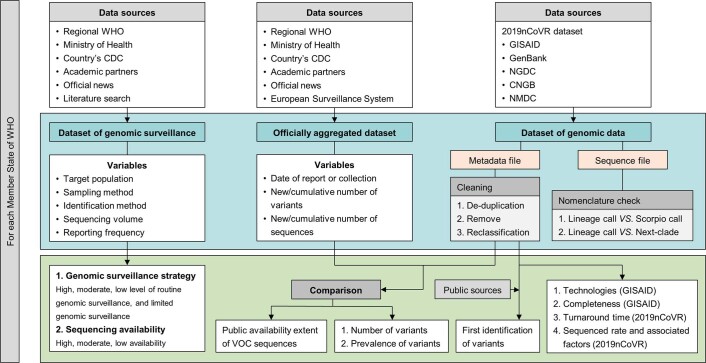
Overall flowchart of data collection and data analysis. GISAID, Global Initiative on Sharing All Influenza Data; NGDC, National Genomics Data Center; CNGB, China National GeneBank; NMDC, National Microbiology Data Center.

We classified genomic surveillance strategies for 118 countries based on predefined criteria (Supplementary Notes and Supplementary Tables 1 and 2), including 78.7% (37/47) of WHO-defined African Region countries, 60.4% (32/53) of European Region countries, 54.3% (19/35) of countries in the Region of the Americas, 57.1% (12/21) of countries in the Eastern Mediterranean Region, 44.4% (12/27) of countries in the Western Pacific Region, and 54.5% (6/11) of countries in the South-East Asia Region (Supplementary Table 3). We downloaded a total of 5.1 million SARS-CoV-2 sequences from public repositories corresponding to samples collected between 1 December 2019 and 31 October 2021. After deduplication efforts across databases and removal of unqualified sequences, there were a total of 4.91 million sequences in 169 countries. To supplement our original search, we downloaded genomic data for the Omicron variant from GISAID to depict the emergence as of 31 December 2021. Additionally, we collected officially aggregated data of variants from 62 countries and extracted data for the first identification of VOCs from 30 countries (Supplementary Tables 4 and 5 and Extended Data Fig. 1).

### SARS-CoV-2 genomic surveillance and sequencing availability

**Fig. 1:**
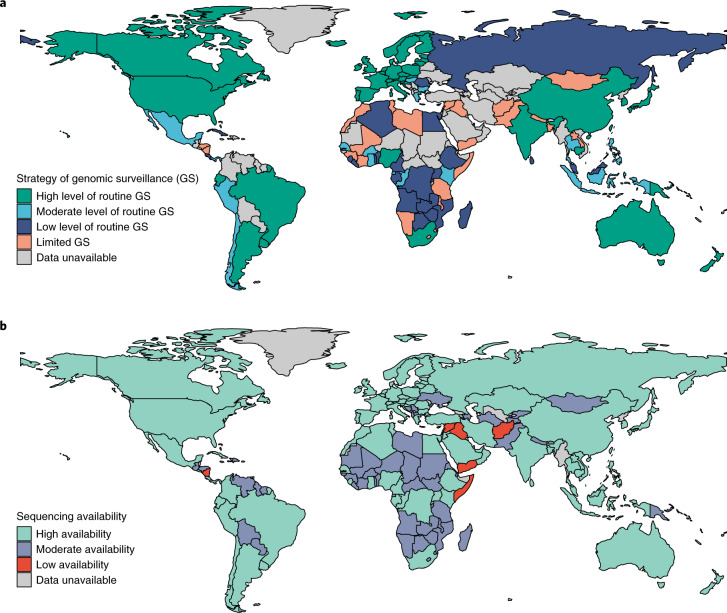
Global SARS-CoV-2 genomic surveillance and sequencing availability. **a**, The global distribution of four strategies for SARS-CoV-2 genomic surveillance. **b**, The global availability of SARS-CoV-2 sequencing. ‘Data unavailable’ include locations that do not belong to the 194 Member States or do not have applicable data. Data shown here are as of 31 October 2021. Administrative boundaries were adapted from the database of Global Administrative Areas (GADM).

We observed marked geographical heterogeneity in genomic surveillance of SARS-CoV-2 across countries. Globally, 38.1% of countries (45) had performed a high level of routine genomic surveillance, 14.4% (17) implemented a moderate level of routine genomic surveillance, 21.2% (25) implemented a low level of routine genomic surveillance, and 26.3% (31) had limited genomic surveillance. The remaining countries (76) had no data on genomic surveillance strategy identified (Fig. 1a). Surveillance diversity across various countries was also reflected in the context of target populations, sampling methods and identification methods (Supplementary Table 3). Specifically, 38 countries randomly selected or used samples from all confirmed cases with sufficient quality for sequencing; 29 countries used the PCR assay to screen probable variants. From the regional perspective, limited genomic surveillance was common in the Eastern Mediterranean Region (83.3%, 10/12), followed by the African Region (27.0%, 10/37), the Region of the Americas (36.8%, 7/19), the South-East Asia Region (33.3%, 2/6) and the Western Pacific Regions (16.7%, 2/12) (Fig. 1a). Among the 172 WHO Member States with data accessible, the sequencing availability of SARS-CoV-2 was high in 96 countries, moderate in 70 countries and low in 6 countries (Fig. 1b).

### Properties of genomic data

**Fig. 2:**
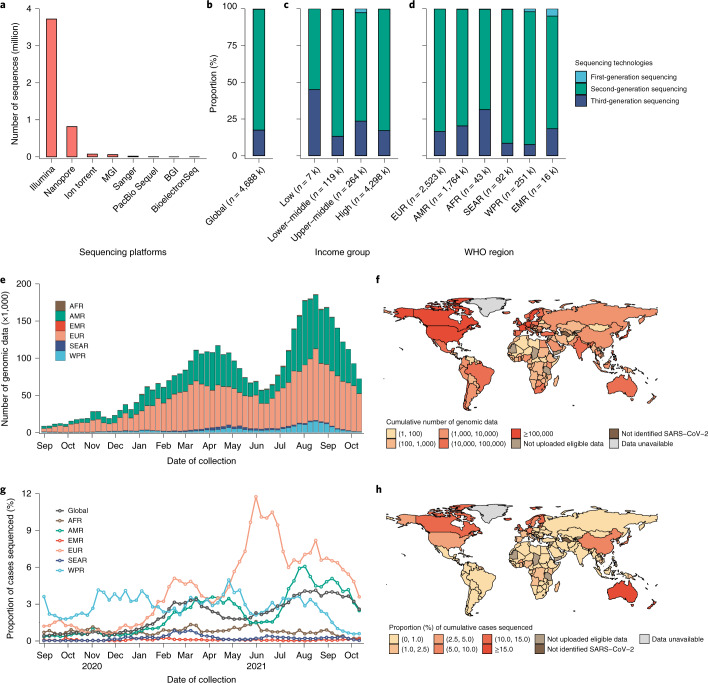
Sequencing technologies and distribution of global publicly deposited genomic data. **a**, Sequencing counts per sequencing platform. Sanger sequencing technology is regarded as a type of sequencing platform in this study. **b**–**d**, The proportions (%) of three types of sequencing technologies (e.g., first-generation sequencing, second-generation sequencing and third-generation sequencing) used globally, by income group and WHO region; we only present the data that were available with sequencing information (*n* = 4.69 million) in the GISAID. **e**, Weekly numbers of publicly deposited SARS-CoV-2 genomic data by region. **f**, Cumulative numbers of publicly deposited SARS-CoV-2 genomic data by country. **g**, Weekly proportions of cases sequenced by region. **h**, Cumulative proportions of cases sequenced by country. The numbers of sequences for the most recent weeks might be incomplete due to time delays between specimen collection and uploading of sequences. The genomic data shown above are eligible, which refer to those with information for the sampling date, sampling country, and lineage available. Data unavailable, include those locations that do not belong to 194 Member States or provide no applicable data. k, stands for 1,000. The range in parentheses in panel **f** and **h** includes the lower bound on the left. AFR, African Region; AMR, Region of the Americas; EMR, Eastern Mediterranean Region; EUR, European Region; SEAR, South-East Asia Region; WPR, Western Pacific Region. Data shown here are as of 31 October 2021. Administrative boundaries were adapted from the GADM database.

**Extended Data Fig. 2:**
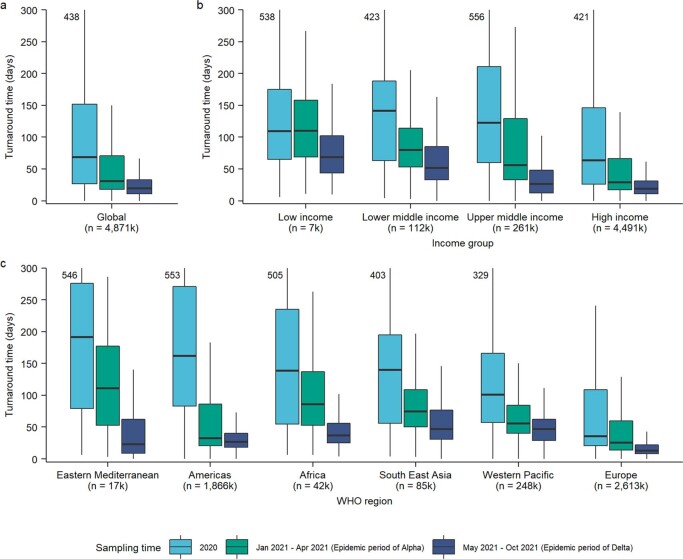
Distribution of turnaround time of SARS-CoV-2 sequences in different time periods. The turnaround time is defined as the time delay between specimen collection and data upload. The lower and upper hinges refer to the 25th and 75th percentiles, respectively; the lower and upper whiskers refer to the smallest values that are greater than or equal to the 1.5 interquartile range from the lower hinge and to the largest values that are further than the 1.5 interquartile range from the upper hinge, respectively. The center line of each boxplot refers to the median value. Outlier points are not shown. We cut off the figure at a y-axis position of 300, and the values of the upper whisker that are beyond 300 are shown next to the bars.

Globally, most sequences were produced on Illumina (*n* = 3,724k, k stands for 1,000) and Nanopore (*n* = 816k) platforms (Fig. 2a). Most sequences were generated using second-generation sequencing technology (82.3%, *n* = 3,856k), with 17.4% (*n* = 817k) from third-generation sequencing and 0.3% (*n* = 15k) from first-generation sequencing (Fig. 2b). The proportion of different sequencing technologies used varied among income groups and WHO regions (Fig. 2c,d). The turnaround time of sequences was shorter in high-income groups (24 days; interquartile range, 14–48 days) and the European Region (18 days; interquartile range, 10–34 days) than in other groups or regions (*P* < 0.0001, *t* test), and turnaround time in all regions shortened as the pandemic progressed (Extended Data Fig. 2).

### Sequencing breadth of SARS-CoV-2 confirmed cases

The European Region (53.8%) and the Region of the Americas (38.2%) uploaded the majority of SARS-CoV-2 sequences to public repositories, with marked intraregion heterogeneity across countries ranging from 2 (Vanuatu) to 1.6 million (United States) as of 31 October 2021 (Fig. 2e,f). High-income countries uploaded 12 times more sequences than non-high-income countries, and the proportion of confirmed cases sequenced in high-income countries (4.36%) was 16 times that of non-high-income countries (0.27%).

Since September 2020, no more than 4.5% of weekly global confirmed cases were sequenced, with a relatively high proportion sequenced in March (3.4%) and early August (4.1%) of 2021. In any week, the African, South-East Asia and Eastern Mediterranean regions sequenced no more than 1.6% of confirmed cases (Fig. 2g). Europe had the highest cumulatively sequenced proportion of 3.4%, followed by the Western Pacific (2.7%), Americas (2.0%), African (0.7%), South-East Asia (0.2%) and Eastern Mediterranean (0.1%) regions. At the country level, higher rates of sequencing were observed in Iceland, Denmark, New Zealand, Australia, Luxembourg, Norway, the United Kingdom, Finland and Canada, all of which had at least 10% of reported cases sequenced as of 31 October 2021. In addition, almost all countries in the African and Eastern Mediterranean regions sequenced less than 2.5% of confirmed cases, except for The Gambia (7.0%), Djibouti (2.7%) and Burkina Faso (2.6%) (Fig. 2h).

**Extended Data Fig. 3:**
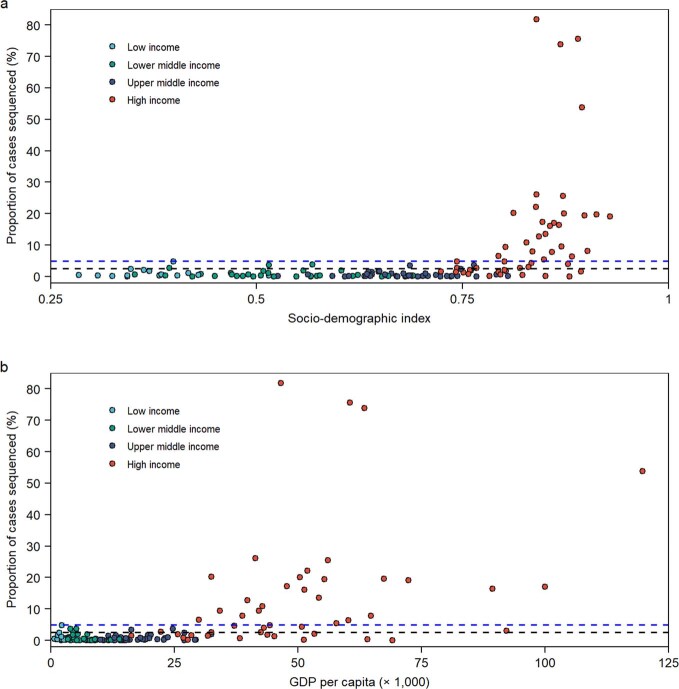
Proportions of cases sequenced in each country plotted against socioeconomic factors. **a**) Proportions of cases sequenced against the sociodemographic index (SDI). The SDI can be divided into five categories: high, high-middle, middle, low-middle, and low. **b**) Proportions of cases sequenced against GDP per capita (unit: international dollars) that are adjusted for purchasing power parity. This analysis is restricted to the time period from May 1, 2021 to September 30, 2021, during which the delta variant began to dominate worldwide. Those countries that deposited fewer than 10 eligible sequences in this period were excluded. The blue and black horizontal dotted lines represent 5.0% and 2.5% of the sequenced percentage, respectively. Note: the sequenced percentage is a rough proxy that is due to the potential non-sharing of some genomic data and underreporting of cases.

We explored the relationship between sociodemographic index (SDI) and sequencing percentage and found that percentage was relatively constant at high-middle to low SDI levels, with a sharp increase in coverage at a high SDI (>0.805; Extended Data Fig. 3a). The relationship between gross domestic product (GDP) per capita and sequencing percentage followed the same general pattern, with a low percentage at low GDP values and a sharp increase in percentage (and variability) at higher levels (Extended Data Fig. 3b).

### Extent of public availability of variant sequences

**Fig. 3:**
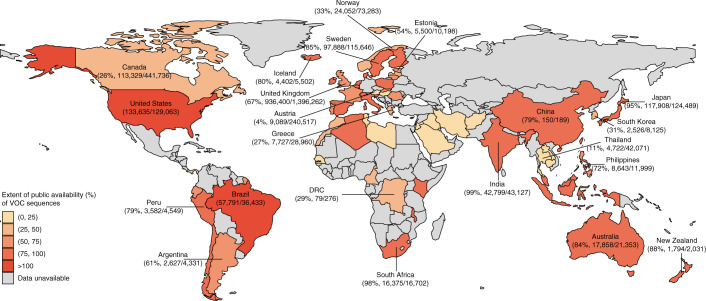
The extent of public availability of VOC sequences in public repositories. In view of the availability of official data, the cumulative numbers of variants in different countries correspond to different time periods, with detailed information contained in Supplementary Table 10. The variant data for China include those that have only been reported for mainland China. The officially reported number of Alpha variants might contain cases that were screened by PCR assays. The extent of public availability over 100% was observed in some countries (United States and Brazil), which was likely due to 1) inconsistent timestamps between the deposited genomic data and aggregated data (we assumed a 3-week collection-to-report time delay for Brazil, but this delay could be longer), 2) incomplete data aggregated in official reporting systems or 3) the number of variants in genomic datasets that may be amplified by multiple sequences that were serially sampled from one patient at longitudinal time points. The sequences in public repositories with no collection dates for the specimens are not included. The Omicron variant was not included in this analysis, as most countries had not yet provided any officially reported data on the Omicron variant at the time of writing. The values beneath the country names indicate the number of cumulative variants during the same period (variants in public repositories/official reported variants). The range in parentheses in the legend includes the lower limit on the left, and includes the upper limit for (75, 100). Data shown here are as of October 31, 2021. Administrative boundaries were adapted from the GADM database. DRC, Democratic Republic of the Congo.

Overall, among countries with aggregated data on the official number of variants, more than one-third (23/62) of countries uploaded less than 50% of their total VOC sequences (Alpha, Beta, Gamma and Delta), and 15 (24.2%) countries uploaded less than 25% of their VOC sequences. Within 33 high-income countries, 9 countries (27.3%) uploaded less than 50% of their total VOC sequences; within 16 low- or lower-middle-income countries, 9 countries (56.3%) uploaded less than 50% of their total VOC sequences (Fig. 3).

**Extended Data Fig. 4:**
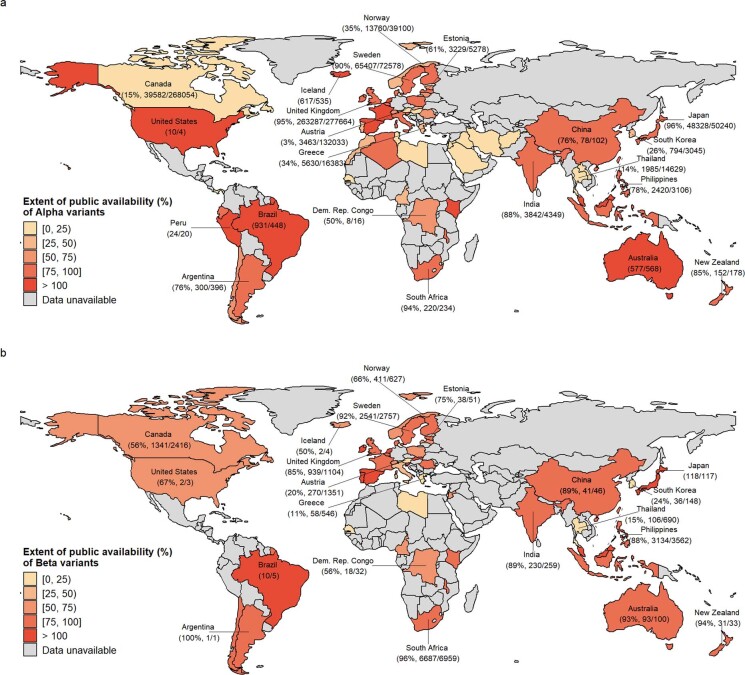
The extent of public availability of Alpha and Beta variant sequences to public repositories. In view of the availability of official data, the cumulative numbers of variants in different countries correspond to different time periods, with the detailed information contained in Supplementary Table 10. The variant data for China include those that have only been reported for mainland China. The officially reported number of alpha variants might contain those that were screened by PCR assays. The extent of public availability over 100% was observed in some countries, which was likely due to 1) inconsistent timestamps between the deposited genomic data and aggregated data (although we assumed a three-week collection-to-report time delay, but this delay could be longer); 2) incomplete data aggregated in official reporting systems; or 3) the number of variants in genomic datasets that may be amplified by multiple sequences that were serially sampled from one patient at longitudinal time points. The sequences in public repositories with no collection dates for the specimens are not included. The values beneath the country names indicate the numbers of cumulative variants during the same period: *variants in public repositories/official reported variants*. Administrative boundaries were adapted from the GADM database.

**Extended Data Fig. 5:**
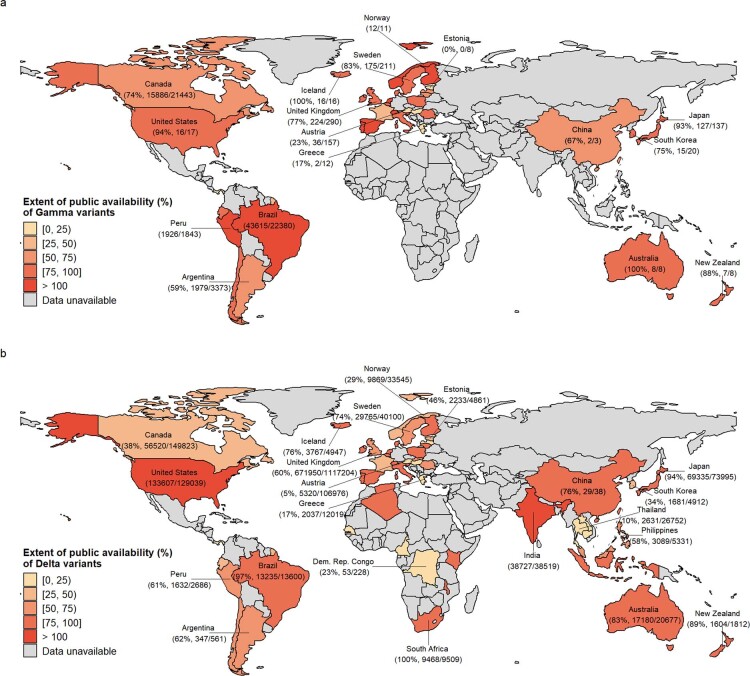
The extent of public availability of Gamma and Delta variant sequences to public repositories. In view of the availability of official data, the cumulative numbers of variants in different countries correspond to different time periods, with the detailed information contained in Supplementary Table 10. The variant data for China include those that have only been reported for mainland China. The extent of public availability over 100% was observed in some countries, which was likely due to 1) inconsistent timestamps between the deposited genomic data and aggregated data (although we assumed a three-week collection-to-report time delay, but this delay could be longer); 2) incomplete data aggregated in official reporting systems; or 3) the number of variants in genomic datasets that may be amplified by multiple sequences that were serially sampled from one patient at longitudinal time points. The sequences in public repositories with no collection dates for the specimens are not included. The values beneath the country names indicate the numbers of cumulative variants during the same period: *variants in public repositories/official reported variants*. Administrative boundaries were adapted from the GADM database.

The extent of public availability of SARS-CoV-2 genomic data varied across countries and variants; less than half of the sequences of Alpha, Beta, Gamma and Delta variants were publicly available in 36.1% (22/61), 17.0% (8/47), 16.2% (6/37) and 33.3% (18/54) countries, respectively (Extended Data Figs. 4,5). However, the result for Alpha might be influenced by SGTF detected via PCR. The extent of public availability of Delta variants across countries ranged from 0.0% (Hungary and Laos) to 100.0%. Seven countries (Austria, Cyprus, Greece, Hungary, Panama, Senegal and Thailand) shared sequences for less than 50% of each VOC. For example, the publicly available proportion of Alpha, Beta and Delta variants in Thailand was 13.6%, 15.4% and 9.8%, respectively, suggesting that more than 80.0% of genomic data related to variants might not be timely uploaded to public databases.

### Quality of related metadata

**Extended Data Fig. 6:**
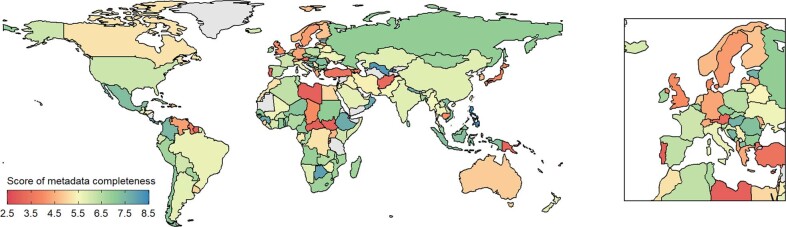
Total scores of metadata completeness. We developed a scoring system to assess the metadata quality of each country based on the metadata completeness of ten key variables, including subnational information, sample strategy, specimen source, sequencing technology, date of collection, sex, age, patient status, vaccinated status, and lineage (the weight of each variable is one point, and the total scores are 10 points). The right panel shows the expanded European region. Administrative boundaries were adapted from the GADM database.

Moreover, incomplete metadata attached to GISAID sequences was common globally, with about 63% of sequences missing demographic information (age and sex), 84% of sequences missing sampling strategy and more than 95% of sequences missing patient-level clinical information (e.g., symptom history, clinical outcome and vaccination status) (Supplementary Tables 6 and 7). High-income regions tended to have more missing information than lower-income regions. For example, in the European Region, less than 25% of sequences had demographic metadata, and only 3% had patient-status clinical information, which is significantly lower than the African, South-East Asia and Eastern Mediterranean regions (*P* < 0.0001, chi-squared test). Furthermore, 94.3% sequences were reported at a subnational geographic resolution. Our quality scoring system for evaluating overall completeness of ten essential variables in metadata indicated a global average completeness level of 5.6/10 points (the average of each country’s scores) and marked heterogeneity between countries, in which the Philippines had the highest quality of 8.4/10 points (Extended Data Fig. 6).

### Earliest identification of SARS-CoV-2 variants

**Fig. 4:**
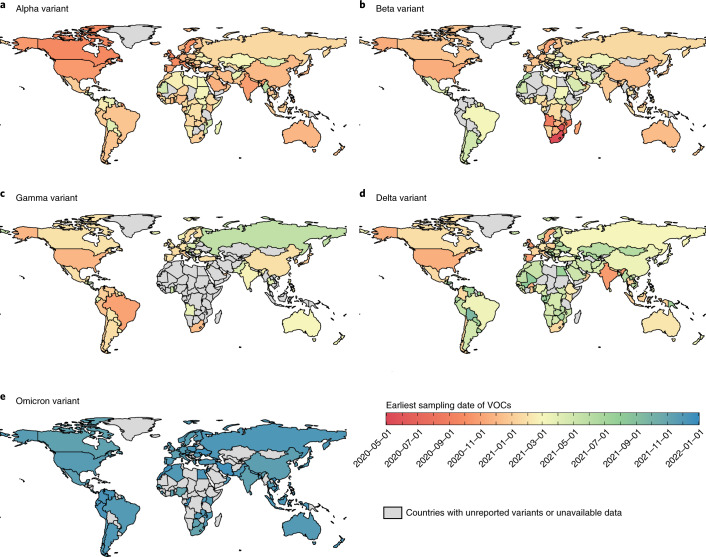
The earliest identification of the Alpha, Beta, Gamma, Delta and Omicron variants in each country. The identification of (**a**) Alpha, (**b**) Beta, (**c**) Gamma, (**d**) Delta, and (**e**) Omicron variants is shown, respectively. If information regarding the earliest sampling dates was unavailable but that of the earliest reporting date was available, then we extrapolated the sampling dates using a fixed 3-week lag from sample collection to reporting. Countries with darker red colors indicate earlier samples, and those with darker blue colors refer to later samples. Data for the Omicron variant are as of 31 December 2021. Administrative boundaries were adapted from the GADM database.

**Fig. 5:**
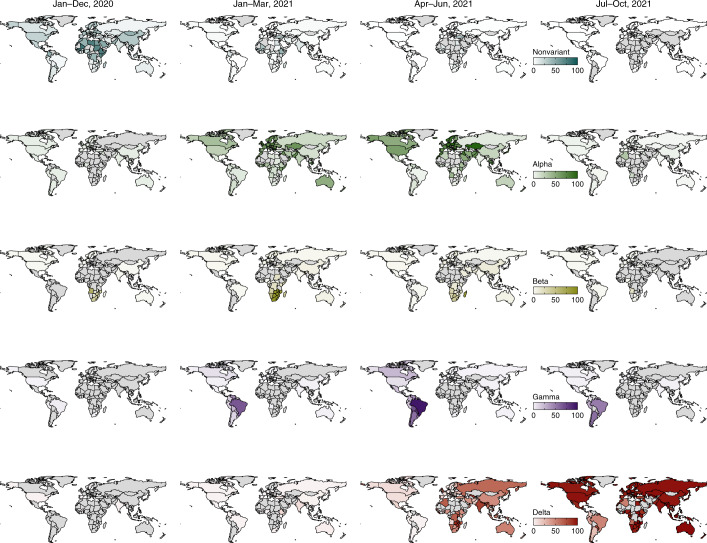
The prevalence and temporal dynamics of nonvariant strains and four SARS-CoV-2 VOCs. The dates shown at the top refer to the date ranges of specimen collection. The prevalence was defined as the proportion of the strain number (nonvariant strains or variants) to the total number of sequences that were generated in the same unit of time. The nonvariant strains include lineages A, A.1, B and B.1; the sublineages of four VOCs are aggregated with the parent lineages. The Omicron variant is not included in this analysis, as the most recent sequencing mainly targeted positive samples of S dropout at the time of writing. The gray areas represent those countries with no COVID-19 epidemic, or no sequencing or no uploads of more than ten eligible genomic data to public repositories in each period. Data shown here are as of 31 October 2021. Administrative boundaries were adapted from the GADM database.

The Alpha variant was first identified in the European Region and then in the Region of the Americas, the Eastern Mediterranean Region and the South-East Asia Region in September-October 2020, followed by the spread to the African Region and Western Pacific Region in November 2020 (Fig. 4a). The earliest publicly available sequenced Beta variant was sampled in Africa in May 2020 and subsequently identified in other regions (Fig. 4b). The Gamma variant has largely remained geographically constrained after it was first identified in Brazil (Fig. 4c). After the first identification of the Delta variant in October 2020 in Southeast Asia, global identification began in January 2021 (Figs. 4d and 5). The Omicron variant was first identified in Africa in November 2021 and subsequently detected in countries of other regions (Fig. 4e)

### Global and regional spread of SARS-CoV-2 variants

**Fig. 6:**
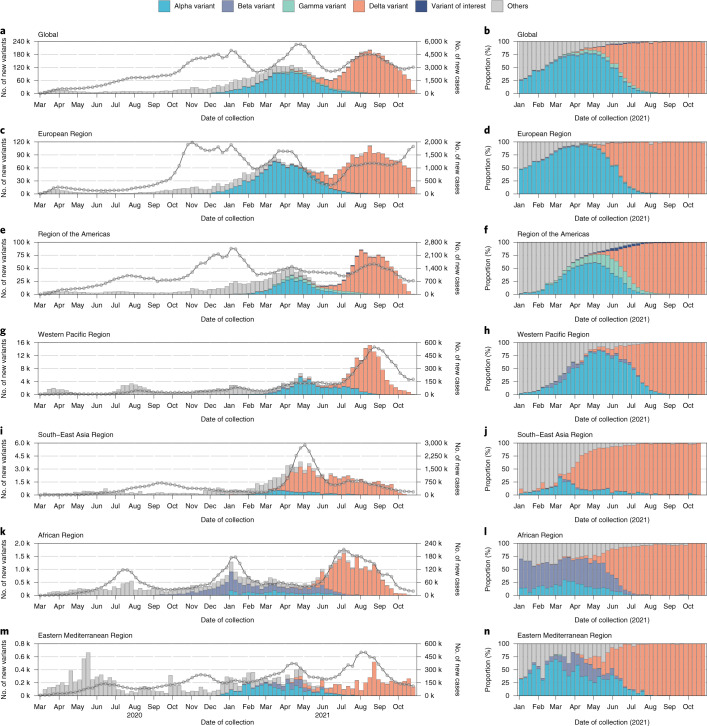
The numbers and proportions of SARS-CoV-2 variants by region and time. Weekly numbers and proportions of SARS-CoV-2 variants in global (**a**–**b**), European Region (**c**–**d**), Region of the Americas (**e**–**f**), Western Pacific Region (**g**–**h**), South-East Asia Region (**i**–**j**), African Region (**k**–**l**), Eastern Mediterranean Region (**m**–**n**). The lines and points in the left panel correspond to the *y* axis on the right. The sublineages of four VOCs were aggregated with the parent lineages; the designated variants of interest include lineages C.37, B.1.621 and their sublineages; other lineages include nonvariant strains and other variants. The data used here were derived from public repositories and aggregated datasets, with priority given to the datasets with the highest number of sequences in a specific week. Data shown here are as of 31 October 2021. k, stands for 1,000.

The number of reported VOC cases dramatically increased until April 2021, with a peak weekly value of about 100,000 VOC cases sequenced, which were mostly Alpha variants (Fig. 6a). Subsequently, another peak of weekly new VOC cases occurred in August 2021, but with a large amount of Delta variants. The number of VOC cases may be an underestimate for the most recent weeks due to collection-to-report time delays. Notably, this increase was also accompanied by an increase in the volume of new sequenced cases and new COVID-19 confirmed cases.

**Extended Data Fig. 7:**
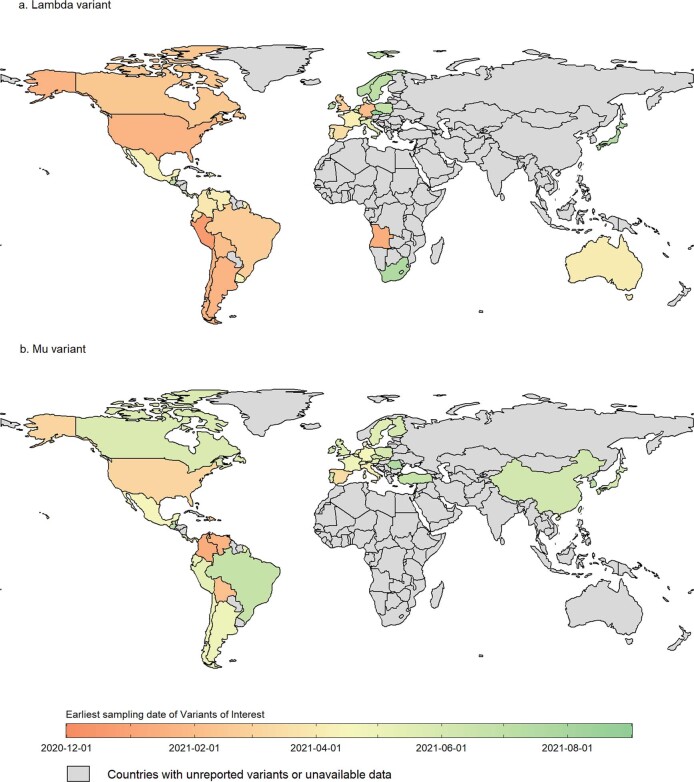
The earliest identification of the Lambda and Mu variants in each country. Administrative boundaries were adapted from the GADM database.

**Extended Data Fig. 8:**
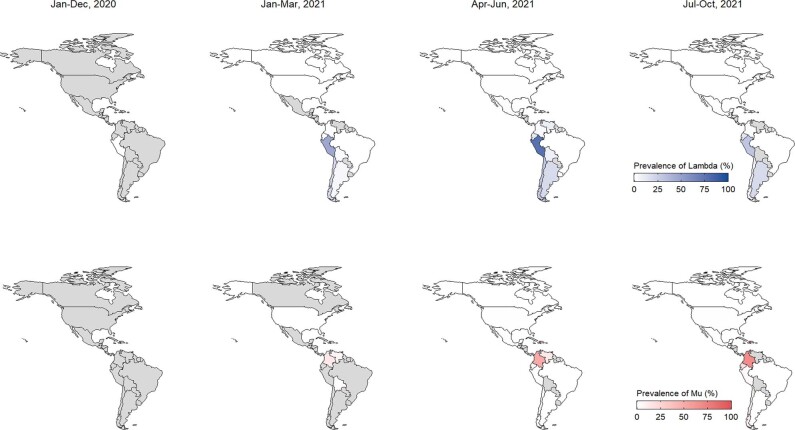
The prevalence and temporal dynamics of the Lambda and Mu variants in the Region of Americas. The countries that deposited more than 10 eligible sequences in each period are included. Since the Lambda and Mu variants are circulating less widely in other regions, only the Region of Americas is presented in the map. Administrative boundaries were adapted from the GADM database.

The global prevalence of nonvariant strains fell to a low level of 0.4% from July to October 2021 compared with 15.2% in 2020 (Fig. 5). Globally, the COVID-19 pandemic was driven by the circulation of the Alpha variant at the start of 2021, with an average prevalence of 51.3% in the first quarter of 2021. Alpha variants continued to outcompete other strains in the second quarter of 2021, accounting for 59.9% of the contemporary lineages (Fig. 5). However, the rapid global rise of the Delta variant began in May 2021, reaching a global prevalence of nearly 98.7% at the end of August 2021 (Fig. 6b). In contrast, Beta and Gamma variants remained at low prevalence (Fig. 5), similar to the variants of interest (VOIs) (Extended Data Figs. 7 and 8). Additionally, the shifting of predominant variants from Alpha to Delta first occurred in Southeast Asia, where the proportion of Delta exceeded 60.0% in April 2021 (Fig. 6c–n).

## Discussion

Our study characterized the global diversity of genomic surveillance strategies and sequencing availability, properties of genomic data, sequenced proportion of SARS-CoV-2 cases, extent of publicly available sequences and the current epidemic trajectory of SARS-CoV-2 variants. We found that genomic surveillance strategies were globally heterogenous, with limited surveillance among many countries in the Eastern Mediterranean Region, African Region and Region of the Americas. Our analysis of publicly deposited SARS-CoV-2 sequences implied that the properties of genomic data were diverse across countries, the cumulative sequenced proportion of cases were low in most countries. Most importantly, our study highlighted that many countries are not sharing genomic data in public repositories. The rapid evolution of SARS-CoV-2 has led to the pervasive spread of the Alpha and Delta variants and highlights the continued threat of SARS-CoV-2 despite the availability of vaccines in many countries.

The diversity of SARS-CoV-2 genomic surveillance between countries is associated with country-specific priorities (e.g., surveillance objectives, targeted monitoring or event- or risk-based sequencing) and available resources. The European Centre for Disease Prevention and Control recommends population-based and/or targeted sampling strategies (for example, imported cases, cluster cases, and potential vaccine escapers) for genomic surveillance^22^, which could provide a more representative estimate of the relative prevalence of variants. Notably, several countries, many of which are classified as low- or lower-middle-income countries by the World Bank, lack genomic surveillance data, likely due to limitations in infrastructure capacity and resources^23^. However, even some countries classified as high-income have suffered from a slow and inconsistent adoption of genomics-based surveillance^24^. Despite gains enabled by the widespread rollout of vaccines in high-income countries, new variants are likely to emerge, as illustrated by the emergence of Omicron variant^12,25^. Enhancing genomic surveillance and sequencing efforts across the globe is an important tool to detect and understand emerging variants. Given the potential for the evolution and circulation of emerging variants in settings with low sequencing capacity^26^, efforts made to increase genomic capacity in such areas, such as the establishment of reference laboratories and networks to provide and/or enhance sequencing services for countries without or with limited established sequencing capacity, may enable improved detection and tracking of emerging variants worldwide.

Importantly, some low- and lower-middle-income countries, such as The Gambia and Nigeria, were observed to have higher proportion of cases sequenced in comparison to other countries in the same group (Supplementary Figs. 1 and 2). The precise factors underlying this discrepancy in sequencing capacity are unclear, but may include low COVID-19 incidence, accessibility to reference sequencing labs, as well as cooperative support from international groups and regional or local public health programs^27,28^. A country’s income level is not the only factor affecting apparent viral sequencing capacity, as low sequencing proportions were also observed in high-income countries (e.g., United Arab Emirates, Kuwait) (Supplementary Fig. 3). These apparent low sequencing proportions might be attributed to high COVID-19 incidence, poor genomic surveillance system, strict regulations governing biospecimens and data sharing, as well as differing norms on public data sharing^24^. However, in general, we note that sequenced proportion is a rough proxy for surveillance capacity, as this can be limited by a lack of sharing of genomic data and underreporting of cases^29^.

The detection of existing and novel variants relies on genomic sequencing, however, sequences only become available to the global community when laboratories have established sequencing capacity, are willing to share, and are legally allowed to upload them. The discrepancies in data sharing were observed in each region, which confirmed that some countries are sequencing but are not uploading. Besides the initial concern about security of genomic data in a centralized repository, the fear of inequitable and incommensurate benefits from data sharing endeavors further dampens each agency’s enthusiasm to upload data, especially in low-income countries^30^. An overarching challenge about how to protect the interests of data depositors to facilitate data sharing needs to be addressed, despite protection mechanisms (for example, user identification, terms of access, data use agreements) provided by platforms such as GISAID^17^. Despite these challenges, improvements to the speed (preferably real-time) and extent of submitting genomic data to publicly available databases is critical for timely public health responses to emerging variants^12^.

The reliability of genomic data as a tool to capture local diversity of variant evolution and spread is dependent on the extent of available metadata from surveillance networks. Different technical, economic, legal, and political barriers may impede the sharing of complete patient-level metadata^31^, with impact across countries of all income levels, as illustrated by our finding of a frequent lack of demographic information shared by high-income countries. Genomic sequences coupled with more complete metadata can maximize the utility of genomic data in rapid scientific discovery during this pandemic, which are valuable for in-depth epidemiological analyses to characterize risk factors, clinical severity, and other public health risk of variants^32–34^. Therefore, it is vital to optimize the sharing of information in a secure and trusted channel in the context of protecting patient anonymity and in accordance of local regulations^35^. Our analysis suggests that the turnaround time between sample collection to deposition of these sequences are decreasing over time, an encouraging sign of progress toward timely sharing of sequencing data. Coupling this with standardizing of metadata may facilitate the consideration of variant spread in the design and development of treatment and prevention strategies by policy-makers^18,32,36,37^.

There are several web-based platforms that have provided up-to-date visualizations of SARS-CoV-2 genomic data and geographic distribution of variants, such as Nextstrain^38^ and outbreak.info^39^. Tools such as these have played invaluable part in dissemination and interpretation of SARS-CoV-2 genomic epidemiology data. However, our analyses extend beyond the information contained in these tools. For example, the currently hosted regional Nextstrain pages for SARS-CoV-2 are, by necessity, subsampled from the 5 million sequences in GISAID to around 3,000–4,000 sequences^38^. Furthermore, we provide the characteristic summaries of global genomic surveillance, together with a suite of characteristics of genomic data.

Our results should be interpreted in view of several limitations. First, the lack of data from some countries limited our global mapping. The data completeness and quality could be impacted by key steps in the surveillance or reporting, including differences in diagnostic criteria, underreporting, delayed reporting and reporting methods. The inconsistent diagnostic criteria of variants might cause sampling bias, especially when adopting nonspecific PCR assays to detect Alpha variant^40^. We did an extensive search to collect multi-source data and chose the aggregated data with a priority to sequencing results rather than PCR-screening results. Also, our efforts to collect and process multi-language data are limited by the accuracy and ability of digital translations. Second, the analysis of global and national spread could be biased as data from public repositories or aggregated dataset are not always representative of the variants circulating in the regions, especially for the regions with relatively limited sequencing capacity or only with investigating outbreak-based events. Therefore, the global diversity of circulating variants may be biased due to the uneven sequencing across the regions and the variety of sampling source of sequences. Third, detailed demographical, epidemiological and clinical information about variant cases is sparse within the current SARS-CoV-2 databases, which limits further epidemiological analyses on outcomes, disease severity, and vaccine efficacy across existing and novel variants.

In conclusion, our study provides a landscape for genomic surveillance, the global breadth of sequencing, properties and public availability of genomic data in the context of repeated emergence of SARS-CoV-2 VOCs. Our findings suggest that global SARS-CoV-2 genomic surveillance strategies and capacity vary considerably, and are limited in some regions. Importantly, our study revealed that in certain countries, a large number of genomes are not available in public databases. To counter the threat of emerging variants, we urge international cooperation in encouraging, incentivizing, and enabling the timely and complete sequencing and sharing of SARS-CoV-2 genomic data in all countries.

## Methods

### Data sources and collection

Through extracting country-specific data from multiple publicly available sources, we built three datasets of genomic surveillance, genomic data deposited in public repositories, and officially aggregated number of variants as of 31 October 2021 (Extended Data Fig. 1).

#### Dataset of genomic surveillance

Each country’s genomic surveillance strategy and sequencing availability was gathered from searches of the websites of regional WHO, the country’s ministry of health and center for disease control, local academic partners and official news, supplemented by a literature search (Supplementary Notes). Data extracted included the overall surveillance strategy, sequencing availability, target population, sampling method, identification method and sequenced volume. Given that the surveillance strategy and density may change with time, we only gathered information on the most recent surveillance strategy (as of 31 October 2021).

#### Dataset of SARS-CoV-2 sequences in public repositories

SARS-CoV-2 metadata files were downloaded from an online coronavirus analysis platform (2019nCoVR)^41^ on 3 November 2021, where has initially merged and deduplicated sequences that deposited in GISAID^17^, GenBank^42^, National Genomics Data Center^43^, National Microbiology Data Center^44^ and China National GeneBank^45^. SARS-CoV-2 sequences was downloaded from the above original public repositories. Considering that a new VOC (Omicron, B.1.1.529) was designated on 26 November 2021, we additionally downloaded all the genomic data of Omicron variants from GISAID on 31 December 2021.

#### Officially aggregated dataset

To gain additional insights regarding the extent of public availability of genomic data of SARS-CoV-2 variants, we extracted country-specific, variant-specific and time-specific aggregated data on the number of SARS-CoV-2 variant cases from official websites, using the same sources as above, except for the literature source. The search was done by either directly locating to the official website for each country or indirectly searching in search engines (Google, Bing or Baidu) using the terms ‘variant’ and country name. To supplement the aggregated data of variants that we collected, we also downloaded the aggregated data with a valid denominator (namely, the number of isolates sequenced is reasonable) from the European Surveillance System. The variables of aggregated dataset included country name, date of report or collection, new or cumulative numbers of different SARS-CoV-2 variant cases and sequenced cases. We only included those countries with such aggregated data available in Supplementary Table 4. Considering that the diagnostic criteria of SARS-CoV-2 variants vary in different countries, the general principle for collecting aggregated data was to give priority to the results based on whole and partial genome sequencing instead of those based on a PCR assay.

For countries noted by WHO as having had VOCs identified but had no data in public repositories, we collected the information about when VOCs were first detected from the country’s ministry of health and official media news, without languages restricted. The search of media news was also done in search engine queries (Google, Bing and Baidu) using combined terms ‘first’ and ‘variant’ and country name. Additional searched data sources are shown in Supplementary Table 5.

All data were entered into a structured database in Microsoft Excel v.2019 by a trained team (coauthors). All recorded data were cross-checked by coauthors.

### Data analysis

We used the variant naming system proposed by WHO, where five VOCs and two VOIs (Lambda and Mu) had been designated as of 27 November 2021 (ref. ^5^). We leveraged our analyses in 194 Member States of WHO and did not integrate data from the overseas territories into that country’s data. Given most countries weekly released aggregated data of variant, most of our analyses were performed on a weekly basis.

#### Genomic surveillance strategy and sequencing availability

To characterize the global landscape of SARS-CoV-2 genomic surveillance, we classified the surveillance strategy of each country into four categories: 1) high level of routine genomic surveillance, 2) moderate level of routine genomic surveillance, 3) low level of routine genomic surveillance and 4) limited genomic surveillance. A high, moderate or low level of routine genomic surveillance was defined as one entity regularly (per month or per week) collects nationwide samples to implement genomic sequencing, coupled with at least 5%, 2.5% or 1.0% of all positive samples sequenced^46–48^ or a certain number of positive specimens sequenced that enables the entity to detect a new variant at a prevalence of 1.0%. 2.5% or 5.0% tailored for this country with a specific range of number of new cases per week (e.g., 501–1,000 or 1,001–2,500) based on a guideline published by the European Centre for Disease Prevention and Control^22^ (Supplementary Notes and Supplementary Table 1), respectively. As we could not identify information on the surveillance strategy for some countries through public sources, we also classified three extra categories according to the public availability/ability of genomic sequencing: 1) high availability, 2) moderate availability and 3) low availability (Supplementary Table 2).

#### Dataset check

We double-checked the duplicates of sequences by targeting those with the same virus name, date of collection and country of collection as the 2019nCoVR repository defined for duplicates^41^; we also treated the same accession ID identified as duplicates. After undergoing deduplication, we kept key-variable-complete (date of collection, country of sampling and assigned lineage) metadata (eligible genomic data) and performed reassignment of thousands of Pango lineages into WHO-designated variants. Detailed cleaning process for data in public repositories is shown in Supplementary Notes. We also examined the potential misclassification of Pango lineage by comparing the consistency of ‘Lineage call’ with ‘Scorpio call’ in Pangolin (v3.1.16) and Nextstrain (Web 1.7.4) nomenclature systems by conveniently selecting about 9,000 sequences sampled in the midpoint of December 2020 and March, June and September 2021; we found the consistent degree reached a level of 100.0% and 99.9%, respectively (Supplementary Table 8). For aggregated data, we collected manually; when the date of sample collection was not available, we assumed a fixed 3-week lag from sample collection to reporting unless country-specific such information was available to inform this extrapolation^49,50^.

#### Properties of genomic data

We analyzed the sequencing technologies and platforms that were used to generate sequences by extracting the sequencing information from metadata in GISAID. We divided sequencing technologies into three types: first-generation sequencing, second-generation sequencing and third-generation sequencing (Supplementary Table 9). We estimated the distribution of turnaround time of all SARS-CoV-2 sequences by the periods of sampling time, income groups and WHO regions. Turnaround time was defined as the time delay between specimen collection and data upload.

#### Sequencing percentage

The sequencing percentage was inferred using the percentage of cumulative positives sequenced as a proxy, which were defined as the ratio of the number of isolates sequenced to the number of confirmed cases in the same unit of time. We explored the associations of sequencing percentage between May and September 2021 (the period when the Delta variant outgrew and dominated other variants) with SDI^51^ and GDP per capita adjusted for purchasing power parity^52^.

#### Extent of public availability of genomic data

Given that not all sequences are uploaded to genomic repositories, we analyzed the extent of public availability of genomic data for those countries with such aggregated data available (Supplementary Table 10). The extent of public availability was defined as the ratio of the cumulative number of variants in public repositories to the official reported number of variants within the same period. Some countries (e.g., United States and Brazil) officially reported the aggregated number of variants, although the agencies acknowledged that the officially aggregated data may be incomplete due to difficulty in capturing these data nationwide. We still included these countries to give a more comprehensive view (Supplementary Table 5). Because the Alpha variant had a characteristic SGTF due to a deletion of amino acids 69 and 70 that can be detected via a widely used PCR assay^53^, we performed this analysis across total VOCs and each VOC.

#### Completeness of released metadata

We evaluated the completeness of variables in released metadata in GISAID by WHO region, income group and country. First, we carefully cleaned these variables to meet the requirement of this analysis. Then, we developed a scoring system to assess the metadata quality of each country based on the metadata completeness degree of ten key variables, including subnational information, sample strategy, specimen source, sequencing technology, date of collection, sex, age, patient status, vaccination status and lineage (where the weight of each variable is one point and the total score is ten points).

#### First identification of variants

We plotted the earliest time when the first VOC or VOI specimen was identified in each country. The earliest identification was defined as the earliest sampling time of sequences deposited in public repositories. If a VOC was identified by WHO but did not have a corresponding sequence in a public repository for one country, then we used the date obtained from other sources (Supplementary Table 5). The sequences with a sampling date earlier than the earliest sample identified in the United Kingdom (for Alpha), South Africa (for Beta), Brazil (for Gamma), India (for Delta), Peru (for Lambda) and Colombia (for Mu), respectively, were not used in analyses.

#### Trajectory of variant spread

We also described the global and regional prevalence trends of variants. The prevalence of a variant was defined as the proportion of the variant number to the sequencing number that was generated in the same period. When multiple data sources were available for one country (e.g., publicly available genomic data and officially aggregated data), then priority was given to the one with the highest number of sequences in a specific week.

Comparison between the values of two groups was made using the *t* test and that between ratios was made using the chi-squared test; differences were considered statistically significant at a two-sided *P* value < 0.05. All statistical analyses and visualizations were done using R (version 4.0.2).

### Reporting Summary

Further information on research design is available in the Nature Research Reporting Summary linked to this paper.

## Online content

Any methods, additional references, Nature Research reporting summaries, source data, extended data, supplementary information, acknowledgements, peer review information; details of author contributions and competing interests; and statements of data and code availability are available at 10.1038/s41588-022-01033-y.

## Supplementary information

Supplementary Information Supplementary Notes, Figures 1–3 and Tables 1–12.

Reporting Summary

Peer Review Information

Supplementary Table Supplementary Tables 3, 7, 11, 12

## Data Availability

Genomic data used in assessing sequencing technology and metadata completeness are available in GISAID (https://www.gisaid.org/). Genomic data used in other analyses are available in 2019nCoVR (https://ngdc.cncb.ac.cn/ncov/release_genome). Officially aggregated dataset of SARS-CoV-2 variants are available on GitHub (https://github.com/zychenfd/Global-landscape-of-SARS-CoV-2-variants). The aggregated data on variants in the European Surveillance System are obtained from https://www.ecdc.europa.eu/en/publications-data/data-virus-variants-covid-19-eueea. Dataset of SARS-CoV-2 genomic surveillance strategies is available in the Supplementary Tables. COVID-19 epidemic data are derived from WHO (https://covid19.who.int/info/). Population data in 2020 were obtained from the United Nations (https://population.un.org/wpp/Download). Administrative boundaries were adapted from the GADM database (https://gadm.org/). All data and analyses will regularly update in the future, and updated figures will be uploaded to GitHub.

## Code availability

The code used to analyze the data is available at GitHub (https://github.com/zychenfd/Global-landscape-of-SARS-CoV-2-variants) and Zenodo (https://zenodo.org/record/5827478#.YdgcCGjP2Ul) (ref. ^54^).
