# Supplementary material for: Global landscape of SARS-CoV-2 genomic surveillance and data sharing
Source: Nat Genet. 2022 Mar 28;54(4):499–507. doi: 10.1038/s41588-022-01033-y (PMC9005350; doi:10.1038/s41588-022-01033-y)
Supplement: Supplementary file 1 — Supplementary Notes, Figures 1–3 and Tables 1–12. [file 41588_2022_1033_MOESM1_ESM.pdf]

nature portfolio

https://doi.org/10.1038/s41588-022-01033-y

Supplementary information

Global landscape of SARS-CoV-2 genomic surveillance and data sharing

In the format provided by the
authors and unedited

Supplementary Information

Global landscape of SARS-CoV-2 genomic surveillance and data sharing

Zhiyuan Chen[1,2], Andrew S. Azman[3,4], Xinhua Chen[1,2], Junyi Zou[1,2], Yuyang Tian[1,2], Ruijia Sun[1,2], Xiangyanyu Xu[1,2], Yani Wu[5], Wanying Lu[1,2], Shijia Ge[1], Zeyao Zhao[1,2], Juan Yang[1,2], Daniel T. Leung[6,7], Daryl B. Domman[8], and Hongjie Yu[1,2,9,10]

Affiliations

1. Department of Infectious Diseases, Huashan Hospital, School of Public Health, Fudan University, Shanghai, China
2. Key Laboratory of Public Health Safety, Fudan University, Ministry of Education, Shanghai, China
3. Department of Epidemiology, Johns Hopkins Bloomberg School of Public Health, Baltimore, MD, USA
4. Institute of Global Health, Faculty of Medicine, University of Geneva, Switzerland
5. School of Public Health, School of Medicine, Shanghai Jiao Tong University, Shanghai, China
6. Division of Infectious Diseases, University of Utah School of Medicine, Salt Lake City, UT, USA
7. Division of Microbiology & Immunology, University of Utah School of Medicine, Salt Lake City, UT, USA
8. Center for Global Health, Department of Internal Medicine, University of New Mexico Health Sciences Center, New Mexico, USA
9. Shanghai Institute of Infectious Disease and Biosecurity, Fudan University, Shanghai, China
10. National Medical Center for Infectious Diseases, Huashan Hospital, Fudan University, Shanghai, China

Corresponding authors: Hongjie Yu, E-mail: yhj@fudan.edu.cn

Table of contents

Supplementary Notes 3

1. Nomenclature of SARS-CoV-2 variants 3
2. Literature search for genomic surveillance strategy 3
3. Classification of genomic surveillance and sequencing availability 3
4. Data cleaning for genomic data and aggregated dataset 4
5. Checking the variant classifications 5
6. Classification of sequencing technologies 6

Supplementary Figures 7

Supplementary Figure 1. Proportions of cases sequenced by income groups 7

Supplementary Figure 2. Proportions of cases sequenced plotted against COVID-19 incidence levels per 100 people in low-income and lower middle-income countries 8

Supplementary Figure 3. Proportions of cases sequenced plotted against COVID-19 incidence levels per 100 people in high-income countries 9

Supplementary Tables 10

Supplementary Table 1. Definitions of different genomic surveillance strategies. 10

Supplementary Table 2. Definitions of the different levels of genomic sequencing availability..11

Supplementary Table 3. Country-specific SARS-CoV-2 genomic surveillance strategy12

Supplementary Table 4. Data sources for aggregated dataset on SARS-CoV-2 variants13

Supplementary Table 5. Additional data sources for the first identification of SARS-CoV-2 variants 15

Supplementary Table 6. Completeness analysis of metadata collected from GISAID dataset by region and income groups. 17

Supplementary Table 7. Completeness analysis of metadata released from GISAID dataset for each country. 18

Supplementary Table 8. Consistency evaluation of variant classifications 19

Supplementary Table 9. Categories of sequencing technologies.....................................20

Supplementary Table 10. The cumulative official number of variants that used for calculating the extent of public availability of genomic data. 21

Supplementary Table 11. An acknowledgement table for all originating laboratories contributed to generating sequences. 24

Supplementary Table 12. An acknowledgement table for all submission laboratories contributed to sharing sequences. 24

Reference 25

Supplementary Notes

1. Nomenclature of SARS-CoV-2 variants

The dynamic SARS-CoV-2 nomenclature system from Phylogenetic Assignment of Named Global Outbreak Lineages (PANGOLIN) adopted a phylogenetic framework to identify new lineages[1], which is the nomenclature that is used for the sequences deposited in public genomic datasets. On 31 May 2021, the WHO announced a new naming system that uses the letters of the Greek alphabet (e.g., Alpha for B.1.1.7) for an easy and coherent application[2] and is hereafter used in our study. Based on a comparative assessment of the phenotypic impact of the SARS-CoV-2 variants, those with significant signals were classified into variants of concern (VOCs) and variants of interest (VOIs).

2. Literature search for genomic surveillance strategy

Data on the genomic surveillance strategy were supplemented by a literature search. We searched PubMed and Europe PMC for peer-reviewed and preprint studies that characterized the country-level strategies for SARS-CoV-2 genomic surveillance from January 1, 2020, to October 31, 2021. The search was performed using the following terms: "SARS-CoV-2", "COVID-19", "sequencing", and "genomic surveillance". Articles published in English and containing information about genomic surveillance and sequencing capability are included. The data from the literature were entered into the structured dataset (Supplementary Table 3).

3. Classification of genomic surveillance and sequencing availability

We classified the surveillance strategy of each country into four categories: 1) high level of routine genomic surveillance, 2) moderate level of routine genomic surveillance, 3) low level of routine genomic surveillance, and 4) limited genomic surveillance. Sequencing 5% of positive samples has been recommended for African countries by the WHO[3] and for European countries by the European Commission[4], and one modelling study demonstrating that sequencing 5% of positive specimens allows the detection of emerging variants at a prevalence level of 0.1% to 1.0%[5]. Therefore, sequencing 5% of positive samples is regarded as the definition of a high level of routine genomic surveillance in our study. However, we considered that countries with high burdens of newly confirmed cases probably struggle to achieve 5% sequencing proportions despite sequencing tens of thousands of positive specimens per week. Therefore, we also adopted an alternative definition for a high level of routine genomic surveillance, namely, it is acceptable if the sequenced sample sizes are sufficient to detect a new variant at a prevalence of 1.0% that is tailored for locations that have a specific range of case numbers per week. The sample size was recommended by the ECDC guideline[6]. For example, if a country has 75,000 cases per week (range: 50,001-

100,000) and needs to detect a new variant at a prevalence of 1.0%, the required sample size for classification as a high level of routine genomic surveillance is 1,500 per week. The classifications of the other three categories were similar (Extended Table 1).

In addition, we also categorised the global sequencing availability, which is classified into three categories: high availability, moderate availability, and low availability. High availability was defined as the category that can collect viral isolates from clinical samples and conduct in-country genomic sequencing. Countries that use regional sequencing networks or are required to ship samples to external labs outside of these countries were placed in the moderate availability category (Extended Table 2). The regional networks in Africa contain several reference laboratories to provide services to countries in their subregions[7]; therefore, the countries where the reference laboratories were located were defined as "high availability", while those countries that are served by the reference laboratories were defined as "moderate availability". If countries had no sequencing capabilities and had low levels of supportive sequencing services from external laboratories and other organizations, we placed them in the low availability category.

4. Data cleaning for genomic data and aggregated dataset

We used the genomic data from GISAID to assess the sequencing technology and metadata completeness since it contained more abundant variables that deposited in the metadata than the data in the 2019nCoVR repository. We used the genomic data from 2019nCoVR to conduct other analyses, since this dataset merged and deduplicated the sequences from multiple genomic repositories.

In terms of cleaning the genomic data, we first double checked the duplicates within the repository and between repositories. Second, we removed the sequences of nonhuman hosts or non-assignments of the PANGO lineage and removed those that did not belong to the 194 Member States. Then, all assigned PANGO lineages were classified into eight categories (e.g., non-variant strains, Alpha, Beta, Gamma, Delta, Lambda, Mu, and other variants) based on the designation system described by the WHO. The non-variant strains included lineages A and B along with the additional lineages A.1 and B.1 that harboured the D614G mutation in the spike protein. The sub-lineages (descendants) of four VOCs and two VOIs were aggregated with the parent lineages. Sequences were also removed if the date of collection was incomplete (e.g., only the year) or sampled before 1 December 2019. When explicit dates were not provided but a month was provided, we selected the middle of the month as the sampling date.

To clean the officially aggregated data, we mainly chose the aggregated results obtained by sequencing rather than screened by PCR assays. The Alpha variant cases included those B.1.1.7 cases with or without the E484K mutation site, as well as the sub-lineages of Q. The Delta variant included lineage B.1.617.2 and its sub-lineages of AY. Regarding the reporting dates in the aggregated dataset, we employed a fixed three-week lag to extrapolate the date of collection[8,9], unless the tailored-made delay information was known for a country.

5. Checking the variant classifications

We performed some analyses to check the consistency of the nomenclatures of variant classifications. First, we checked the consistency between "Lineage call" and "Scorpio call" for the same sequences. We conveniently selected some sequence samples that were collected at four timepoints (midpoints of December 2020, March 2021, June 2021, and September 2021), given the handling power of the online nomenclature systems. Then, we ran those sequences by using the Pangolin tool (https://pangolin.cog-uk.io/) to obtain the assigned "Lineage call" and "Scorpio call" for each sequence. We selected some specific variants (including Alpha, Beta, Gamma, Delta, Lambda, Mu, Kappa, Epsilon, Theta, Iota, Eta, and EU1) after undergoing classification by each nomenclature system to calculate the assignment consistencies. The sequences without assigned a variant designation were not included in the consistency analysis. For example, if a sequence was assigned to "none" by "Lineage call" but was assigned to "alpha-like" by "Scorpio call", we removed this sequence to calculate the consistency. As a result, there was a high consistency (100.0%) between "lineage call" and "Scorpio call" (Supplementary Table 8).

Second, we also evaluated the degree of consistency between Pangolin and Nextstrain nomenclature systems for the same sequences downloaded as above. We ran these same sequences in the Pangolin tool (https://pangolin.cog-uk.io/) and Nextclade (https://clades.nextstrain.org/results) to assign lineage or clade classifications, respectively. Similarly, we selected those specific variants after undergoing classification by each nomenclature system to calculate assigned consistencies. Finally, we found that the degree of consistency reached 99.9% (Supplementary Table 8).

Although the nomenclature is sometimes changing at a rapid pace of the continuous updates and improvements of the Pangolin tools, incorrect lineage assignments still occasionally occur, especially when there is a certain proportion of ambiguous sites[10]. However, the genomic dataset (e.g., GISAID) assigns a lineage designation only for sequences that have less than 5% of ambiguous nucleotide sites in the coding regions[10]; therefore, the proportion of misallocations is expected to be very low.

6. Classification of sequencing technologies

We carefully cleaned the sequencing technical information of each sequence in GISAID and divided the sequencing technologies into three types: first-generation sequencing, second-generation sequencing, and third-generation sequencing (Supplementary Table 9). When only the information about the sequencing assay/panel is available, and it is compatible for second generation sequencing; we considered those sequences were generated from second generation sequencing. We also assumed that all Illumina® platforms generating SARS-CoV-2 sequences are categorized as second-generation sequencing[11].

Supplementary Figures

Supplementary Figure 1. Proportions of cases sequenced by income groups

Each dot represents one country that deposited at least 10 sequences from May 1, 2021 to September 30, 2021. The green dots refer to those low-income and lower middle-income countries with more than or equal to a 2.5% proportion of cases sequenced, which include Papua New Guinea, Nigeria, Congo. Rep., and The Gambia, while the red dots refer to those 14 high-income countries (Brunei Darussalam, Chile, Andorra, Greece, Israel, Malta, The Bahamas, Barbados, Trinidad and Tobago, Bahrain, Kuwait, Oman, United Arab Emirates, Seychelles) with less than a 2.5% proportion of infections sequenced. The blue and black horizontal dotted lines represent 5.0% and 2.5% of the sequenced percentages, respectively. Note: the sequenced percentage is a rough proxy due to the potential non-sharing of some genomic data and underreporting of confirmed cases. Administrative boundaries were obtained from the database of Global Administrative Areas (GADM).

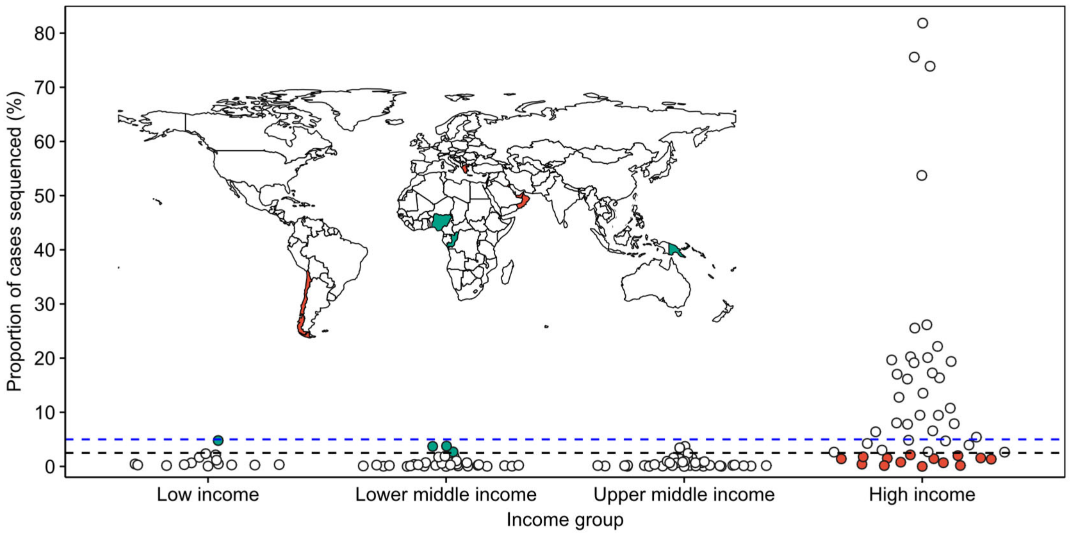

Supplementary Figure 2. Proportions of cases sequenced plotted against COVID-19 incidence levels per 100 people in low-income and lower middle-income countries

The blue horizontal dotted lines represent 1.5% of the sequenced percentage. We added the country name where the proportions of cases sequenced were more than 1.5%. Among the low- and lower middle-income countries, high sequencing percentages are mainly distributed in locations with low COVID-19 incidence rates. In addition, the African reference laboratories of SARS-CoV-2 genomic surveillance are partly located in Nigeria, Kenya, The Gambia, and Ghana, which may be another potential reason why these countries perform well.

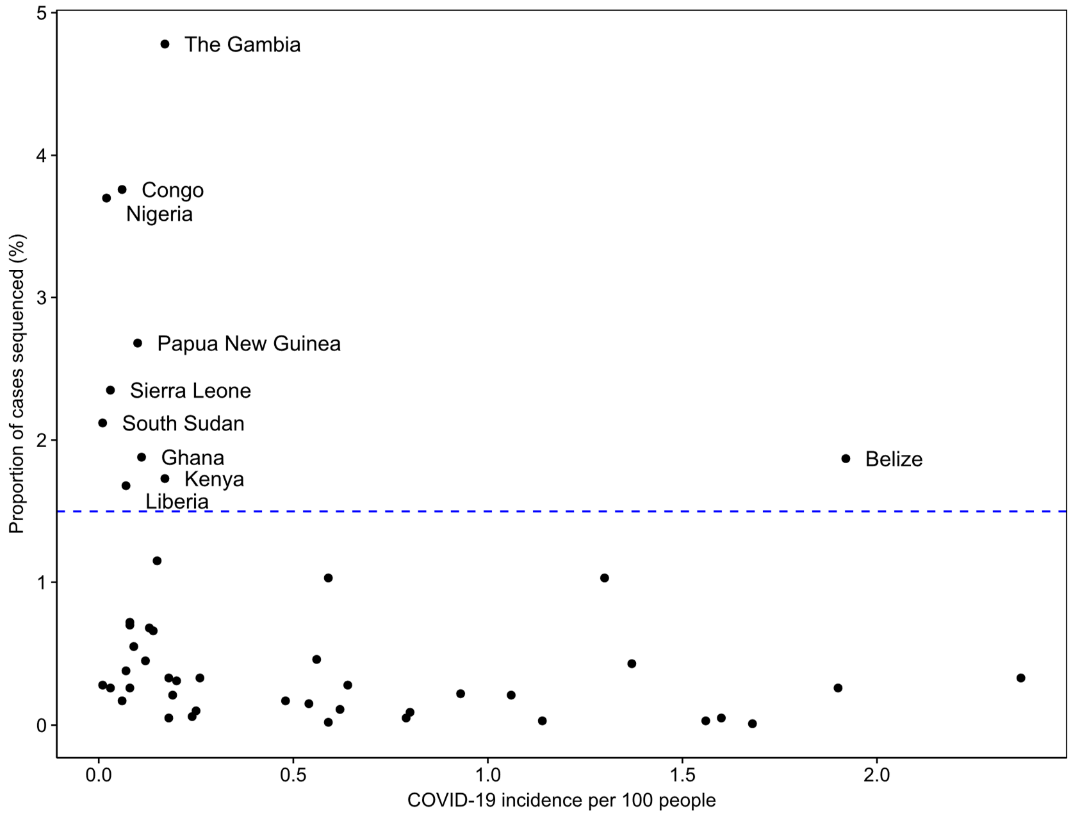

Supplementary Figure 3. Proportions of cases sequenced plotted against COVID-19 incidence levels per 100 people in high-income countries

The blue horizontal dotted lines represent 1.5% of the sequenced percentage. Among the high-income countries, high sequencing percentages are also mainly distributed in locations with low COVID-19 incidence rates. In addition, some countries have low extent of public availability of genomic data (e.g., Greece), which may be another potential reason why these countries seem to perform not well (as this analysis was based on the publicly available genomic data in public repositories).

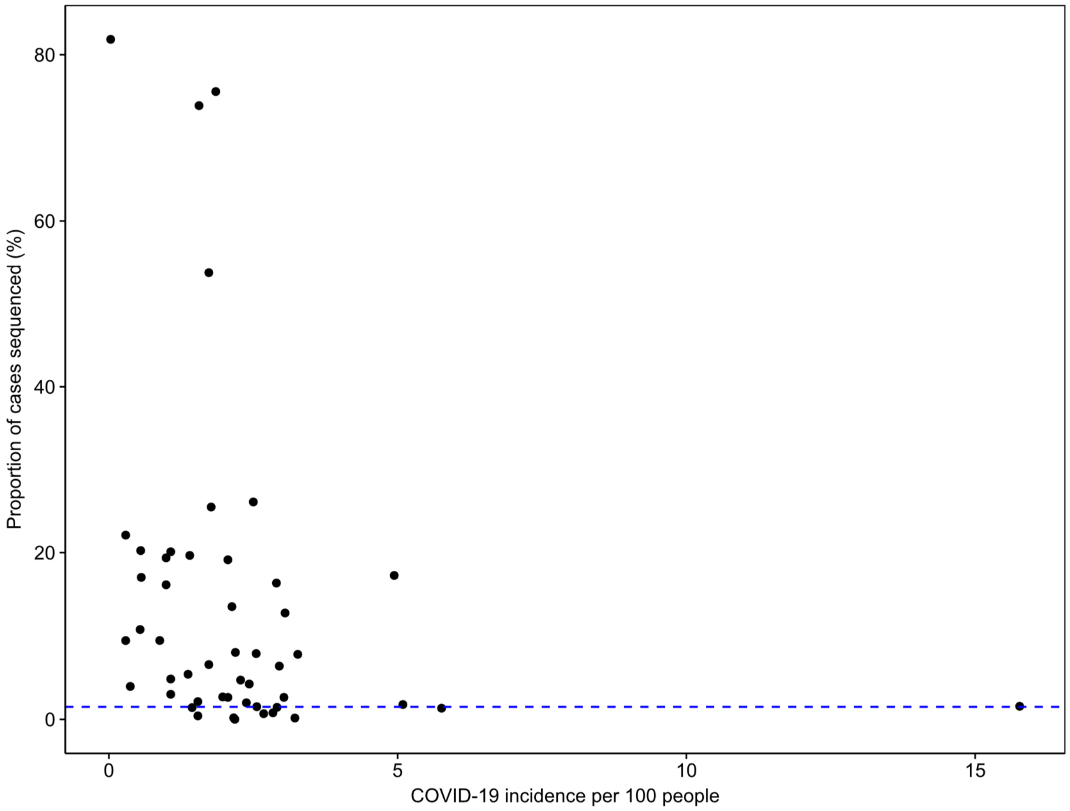

Supplementary Tables

Supplementary Table 1. Definitions of different genomic surveillance strategies.

**Genomic surveillance strategy**	**Definition**		
	**Strategy**	**Sequencing volume (meet one of them)**	
		**Proportion**	**Smallest sequenced number per week**
High level of routine genomic surveillance	Regularly (per month or per week) collect nationwide samples to implement genomic sequencing	At least 5.0% of positive samples sequenced	A certain number of positive specimens sequenced that enables entity to detect a new variant at a prevalence of 1.0% tailored for this country with a specific range of number of new cases per week (e.g., 501-1000, or 1001-2500) based on a guideline published by European CDC[6]
Moderate level of routine genomic surveillance		2.5-5.0% of positive samples sequenced	A certain number of positive specimens sequenced that enables entity to detect a new variant at a prevalence of 2.5% tailored for this country with a specific range of number of new cases per week (e.g., 501-1000, or 1001-2500) based on a guideline published by European CDC[6]
Low level of routine genomic surveillance		1.0-2.5% of positive samples sequenced	A certain number of positive specimens sequenced that enables entity to detect a new variant at a prevalence of 5.0% tailored for this country with a specific range of number of new cases per week (e.g., 501-1000, or 1001-2500) based on a guideline published by European CDC[6]
Limited genomic surveillance	Conduct genomic sequencing, but with limited sequence capacity		

Supplementary Table 2. Definitions of the different levels of genomic sequencing availability.

Classification	Definition
High availability	Be able to collect viral isolates from clinical samples and conduct in-country genomic sequencing.
Moderate availability	Be able to collect viral isolates from clinical samples, but the process of genomic sequencing needs extra supports from external sequencing labs, including the following scenarios: 1) samples need to be shipped to the regional reference labs or high-income countries for sequencing; 2) purchase the commercial kits for the detect SARS-CoV-2 variants or get a donation of that; 3) establish the sequencing laboratory with the support from others during the pandemic of COVID-19.
Low availability	Lack of sequencing capability and have little supportive sequencing services from the external labs.

Supplementary Table 3. Country-specific SARS-CoV-2 genomic surveillance strategy

[table in Excel file]

Supplementary Table 4. Data sources for aggregated dataset on SARS-CoV-2 variants

Country name	Main sources
**European Region**	
Switzerland	Federal Office of Public Health (https://www.covid19.admin.ch/en/epidemiologic/virus-variants)
Sweden	The Swedish Public Health Agency (https://www.folkhalsomyndigheten.se/smittskydd-beredskap/utbrott/aktuella-utbrott/covid-19/statistik-och-analyser/sars-cov-2-virusvarianter-av-sarskild-betydelse/)
United Kingdom	Public Health England (https://www.gov.uk/government/publications/covid-19-variants-genomically-confirmed-case-numbers)
Spain	Ministry of Health, Consumption and Social Welfare (https://www.mscbs.gob.es/profesionales/saludPublica/ccayes/alertasActual/nCov/situacionActual.htm)
Luxembourg	National Health Laboratory (https://lns.lu/departement/microbiologie/revilux/)
Netherlands	National Institute for Public Health and the Environment (https://www.rivm.nl/en/coronavirus-covid-19/virus-sars-cov-2/variants)
Norway	The National Institute of Public Health (https://www.fhi.no/en/publ/2020/weekly-reports-for-coronavirus-og-covid-19/)
Ireland	Health Protection Surveillance center (https://www.hpsc.ie/a-z/respiratory/coronavirus/novelcoronavirus/surveillance/summaryofcovid-19virusvariantsinireland/)
Denmark	Danish Covid-19 Genome Consortium (https://www.covid19genomics.dk/statistics)
Austria	The Austrian Agency for Health and Food Safety (AGES) (https://www.ages.at/themen/krankheitserreger/coronavirus/sars-cov-2-varianten-in-oesterreich/)
France	Public health of France (https://www.santepubliquefrance.fr/etudes-et-enquetes/enquetes-flash-evaluation-de-la-circulation-des-variants-du-sars-cov-2-en-france)
Belgium	Sciensano (https://datastudio.google.com/embed/reporting/c14a5cfc-cab7-4812-848c-0369173148ab/page/urrUC)
Slovenia	National Laboratory for Health, Environment and Food (https://www.nlzoh.si/objave/?tag=mikrobiologija)<br>Institute for Microbiology and Immunology (IMI) of the Medical Faculty in Ljubljana (http://www.imi.si/o-institutu/novice)
Other European countries	European CDC (TESSy) (https://www.ecdc.europa.eu/en/publications-data/data-virus-variants-covid-19-eueea)
**Region of Americas**	
Canada	CTV New.ca's variant tracker (https://www.ctvnews.ca/health/coronavirus/tracking-variants-of-the-novel-coronavirus-in-canada-1.5296141)
United States	United States CDC (https://covid.cdc.gov/covid-data-tracker/#variant-proportions)
Argentina	1. Ministry of Health (https://www.argentina.gob.ar/coronavirus/informes-diarios/vigilancia-genomica)<br>2. Argentine Interinstitutional SARS-CoV-2 Genomic Project (for four provinces) (http://pais.qb.fcen.uba.ar/files/reportes/pais-reporte28.pdf)

Brazil	Ministry of Health<br>(https://www.gov.br/saude/pt-br/media/pdf/2021)
Peru	Ministry of Health<br>(https://web.ins.gob.pe/es/covid19/secuenciamiento-sars-cov2)
Chile	The Government of Chile<br>(https://www.gob.cl/coronavirus/cifrasoficiales/#datos)
Ecuador	Ministry of Public Health<br>(https://www.salud.gob.ec/se-reporta-disminucion-de-casos-delta-en-ecuador/)
Panama	Instituto Conmemorativo Gorgas<br>(http://www.gorgas.gob.pa/informes-de-vigilancia-molecular-sars-cov-2-panama/)
**South-East Asia Region**	
India	Institute of Genomics and Integrative Biology<br>(http://clingen.igib.res.in/covid19genomes/)
Indonesia	Health Research and Development Agency<br>(https://www.litbang.kemkes.go.id/)
Thailand	Department of Medical Sciences Ministry of Health<br>(https://www3.dmsc.moph.go.th/post-group/10)
**Western Pacific Region**	
Australia	1. The CDGN VOC Taskforce<br>(https://www.cdgn.org.au/variants-of-concern)<br>2. Ministry of Health<br>(https://www1.health.gov.au/internet/main/publishing.nsf<br>/Content/1D03BCB527F40C8BCA258503000302EB/$File)
South Korea	Korea Disease Control and Prevention Agency<br>(http://www.kdca.go.kr/board/board.es?mid=a30501000000&bid=0031&cg_code=C05)
Philippines	Department of health<br>(https://doh.gov.ph/press-releases)
Malaysia	Ministry of Health<br>(http://covid-19.moh.gov.my/semasa-kkm/2021)
New Zealand	Ministry of Health<br>(https://www.health.govt.nz/our-work/diseases-and-conditions/<br>covid-19-novel-coronavirus/covid-19-resources-and-tools/covid-19-science-news)
Japan	Ministry of Health, Labour and Welfare & National institute of infectious diseases<br>(https://www.mhlw.go.jp/stf/seisakunitsuite/newpage_00054.html)
Laos	WHO Regional for the WPR<br>(https://www.who.int/laos/emergencies/covid-19-in-lao-pdr/situation-reports)
Cambodia	WHO Regional for the WPR<br>(https://www.who.int/cambodia/emergencies/covid-19-response-in-cambodia/situation-reports)
China	China CDC (unpublished work)
**African Region**	
Cameroon	Anadolu Agency<br>(https://www.aa.com.tr/en/africa/cameroon-on-alert-after-detection-of-alpha-beta-delta-strains-of-covid-19/2333205)
African countries	Africa CDC<br>(https://africacdc.org/institutes/africa-pathogen-genomics-initiative/)
**Eastern Mediterranean Region (EMR)**	
Countries in the EMR	WHO Regional for the EMR<br>(http://www.emro.who.int/health-topics/corona-virus/situation-reports.html)

Supplementary Table 5. Additional data sources for the first identification of SARS-CoV-2 variants

Country name	Source type	Date type (variants)	Date	Main sources
**European Region**				
Belarus	Media news	Date of report (Delta)	2021/6/23	https://apa.az/en/xeber/cis-countries-news/belarus-reports-first-case-of-covid-19-delta-variant-ministry-of-health-says-352358
Cyprus	Media news	Date of report (Beta)	2021/5/19	http://www.xinhuanet.com/english/2021-05/20/c_139956862.htm
		Date of collection (Delta) [a]	2021/4/22	https://in-cyprus.philenews.com/sequencing-shows-increase-of-delta-variant-in-cyprus/
Estonia	Media news	Date of report (Gamma)	2021/5/27	https://news.err.ee/1608226756/brazilian-indian-coronavirus-strains-recorded-in-estonia
Hungary	Media news	Date of report (Beta)	2021/2/26	https://hungarytoday.hu/coronavirus-south-african-variant-hungary-muller/
		Date of report (Delta)	2021/6/30	https://xpatloop.com/channels/2021/06/nine-delta-coronavirus-variant-cases-identified-in-hungary.html
Kazakhstan	Media news	Date of report (Beta)	2021/3/30	https://astanatimes.com/2021/03/kazakh-capital-city-announces-new-restrictions-as-cases-surge/
		Date of report (Delta)	2021/6/22	https://www.reuters.com/business/healthcare-pharmaceuticals/kazakhstan-detects-delta-variant-central-asia-braces-third-covid-wave-2021-06-22/
Kyrgyzstan	Media news	Date of collection (Alpha)	2021/3/26	http://med.kg/en/news/4380-four-covid-19-variants-are-circulating-in-kyrgyzstan.html
		Date of collection (Beta)	2021/3/26	
Ukraine	Media news	Date of report (Beta)	2021/4/1	https://www.reuters.com/article/health-coronavirus-ukraine-idUSS8N2JP00X
Uzbekistan	Media news	Date of collection (Alpha)	2021/1/23	https://for.kg/news-687161-en.html
**Region of Americas**				
Antigua and Barbuda	Media news	Date of collection (Beta)	2021/4/20	https://caribbean.loopnews.com/nl/node/550384
Bolivia	Media news	Date of report (Alpha)	2021/4/22	https://brazilian.report/liveblog/2021/04/22/bolivia-british-coronavirus-variant/
		Date of collection (Delta) [a]	2021/9/15	https://correodelsur.com/sociedad/20211029_salud-detecta-cuatro-casos-de-la-variante-delta-del-covid-19-en-la-paz.html
Cuba	Media news	Date of report (Alpha)	2021/1/22	https://www.miamiherald.com/news/nation-world/world/americas/cuba/article248782480.html
		Date of report (Beta)	2021/1/26	
		Date of report (Delta)	2021/5/15	https://www.radiohc.cu/en/noticias/nacionales/267249-delta-variant-of-sars-cov-2-is-present-throughout-cuba
Dominica	Media news	Date of report (Delta)	2021/8/31	https://www.jamaicaobserver.com/latestnews/Dominica_reports_cases_of_Delta_variant
El Salvador	Media news	Date of report (Delta)	2021/7/31	https://www.reuters.com/world/americas/el-salvador-detects-first-case-covid-19-delta-variant-health-minister-2021-07-31/
Saint Kitts and Nevis	Media news	Date of collection (Delta)	2021/7/28	https://today.caricom.org/2021/08/19/delta-variant-confirmed-in-st-kitts-and-nevis/
Uruguay	Media news	Date of collection (Alpha) [b]	2020/12/20	https://www.republica.com.uy/hallaron-la-variante-britanica-del-virus-sars-cov-2-en-uruguay-id810761/
	Media news	Date of report (Beta)	2021/7/17	https://primetimezone.com/world/uruguay-registers-first-cases-of-delta-and-beta-variant-of-covid-19-prime-time-zone/
	Media news	Date of report (Delta)	2021/7/17	
Saint Vincent and the Grenadines	Media news	Date of collection (Gamma)	2021/8/14	https://www.284media.com/regional/2021/09/13/gamma-variant-confirmed-in-st-vincent-the-grenadines/
	Official data	Date of collection (Delta)	2021/7/27	http://health.gov.vc/health/index.php/c/1708-the-detection-of-a-variant-of-a-concern-in-saint-vincent-and-the-grenadines
**Western Pacific Region**				

Brunei [a]	Media news	Date of report (Alpha)	2021/2/15	http://www.moh.gov.bn/Lists/Latest%20news/NewDispForm.aspx?ID=816
Laos	Media news	Date of report (Delta)	2021/6/30	https://www.thestar.com.my/aseanplus/aseanplus-news/2021/07/01/laos-records-first-cases-of-delta-variant
Mongolia	Media news	Date of report (Alpha)	2021/4/23	http://theubposts.com/uk-variant-of-covid-19-reported-in-mongolia/
		Date of report (Delta)	2021/7/1	http://www.xinhuanet.com/english/asiapacific/2021-07/02/c_1310038709.htm
Vietnam [b]	Media news	Date of collection (Beta)	2020/12/19	https://e.vnexpress.net/news/news/vietnam-detects-1st-case-of-south-african-coronavirus-variant-infection-4229589.html
**African Region**				
Algeria	Media news	Date of collection (Alpha)	2021/2/19	https://www.aps.dz/sante-science-technologie/118228-covid-19-deux-cas-du-variant-britannique-decouverts-en-algerie
Cameroon	Official data	Date of report (Delta) [a]	2021/5/15	https://reports.unocha.org/en/country/cameroon/card/1D7RNQ8SRM/
Cape Verde	Media news	Date of report (Delta)	2021/8/9	https://inforpress.cv/covid-19-ministry-of-health-confirms-circulation-of-the-english-variant-in-cabo-verde/
Liberia	Official data	Date of report (Alpha)	2021/3/2	https://africacdc.org/download/outbreak-brief-59-coronavirus-disease-2019-covid-19-pandemic/
Mauritania [a]	Media news	Date of report (Alpha)	2021/5/15	https://fews.net/west-africa/mauritania/remote-monitoring-report/august-2021
		Date of report (Beta)	2021/5/15	
		Date of report (Delta)	2021/6/15	
Sao Tome and Principe	Media news	Date of report (Delta)	2021/7/27	https://www.voaportugues.com/a/variante-delta-da-covid-19-detectada-em-s%C3%A3o-tom%C3%A9-e-pr%C3%ADncipe-/5982390.html
Eastern Mediterranean Region				
Iran	Official data	Date of report (Alpha)	2020/12/30	http://www.emro.who.int/images/stories/coronavirus/13.pdf?ua=1
Libya	Official data	Date of report (Beta)	2021/3/17	http://www.emro.who.int/images/stories/coronavirus/12.pdf?ua=1
Pakistan	Official data	Date of report (Gamma)	2021/5/2	http://www.emro.who.int/images/stories/coronavirus/12.pdf?ua=1
Saudi Arabia	Official data	Date of report (Alpha)	2020/12/14	http://www.emro.who.int/images/stories/coronavirus/13.pdf?ua=1
		Date of report (Delta)	2021/5/25	http://www.emro.who.int/images/stories/coronavirus/12.pdf?ua=1

[a] Exact date was not reported. Here we used the 15th of each month to replace those with only month information available and adopted the median days to replace those only with date range available.

[b] Arrival time from other countries. Here we assumed that the sample will be immediately collected after landing.

Supplementary Table 6. Completeness analysis of metadata collected from GISAID dataset by region and income groups.

	Total sequences (N)	National geography [a]	Subnational geography [a]	Sample strategy	Specimen source	Sequencing method	Date of collection	Age	Sex	Patient status [b]	Vaccinated status [c]	Lineage
Global												
Total	4736168	100.0%	94.3%	16.1%	33.0%	99.0%	97.1%	37.1%	37.0%	3.6%	0.3%	97.9%
WHO region												
AFR	44004	100.0%	92.6%	28.3%	56.6%	98.6%	97.6%	87.3%	89.7%	14.1%	0.3%	96.5%
AMR	1781787	100.0%	99.8%	35.0%	58.2%	99.0%	97.2%	56.9%	57.9%	2.8%	0.3%	98.8%
EMR	15848	100.0%	75.4%	7.7%	63.3%	99.1%	94.9%	62.7%	64.4%	18.9%	0.9%	93.7%
EUR	2548298	100.0%	92.8%	4.6%	16.5%	99.0%	97.2%	21.7%	20.5%	2.9%	0.2%	97.5%
SEAR	92183	100.0%	98.1%	1.7%	41.2%	99.6%	91.5%	79.7%	82.2%	8.7%	2.0%	95.8%
WPR	254048	100.0%	70.0%	3.0%	13.0%	98.6%	97.4%	27.0%	28.6%	11.1%	0.1%	97.8%
Income group [d]												
Low	6541	100.0%	80.5%	10.7%	63.0%	99.4%	92.8%	72.9%	74.7%	18.4%	0.0%	98.5%
Lower middle	119682	100.0%	94.2%	10.1%	48.2%	99.6%	92.8%	82.1%	82.6%	19.4%	1.5%	96.2%
Upper middle	266268	100.0%	73.7%	5.5%	54.5%	99.1%	98.3%	57.9%	61.2%	20.8%	0.8%	96.3%
High	4343677	100.0%	95.5%	16.9%	31.2%	99.0%	97.1%	34.5%	34.2%	2.0%	0.2%	98.1%

[a] The completeness of geographic information (national or subnational) reported in metadata file.

[b] The completeness of information about patient status, includes the symptomatic history, clinical severity or outcome, etc.

[c] The completeness of vaccinated status, includes whether vaccinated or not, vaccinated platform, vaccinated dose, or vaccinated time, etc.

[d] The newest categories of income group for each country are obtained from the World Bank (https://datahelpdesk.worldbank.org/knowledgebase/articles/906519-world-bank-country-and-lending-groups).

Abbreviations: AFR, African Region; AMR, Region of Americas; EMR, Eastern Mediterranean Region; EUR, European Region; SEAR, South-East Asia Region; WPR, Western Pacific Region. The data used is as of October 31, 2021.

Supplementary Table 7. Completeness analysis of metadata released from GISAID dataset for each country.

[table in Excel file]

Supplementary Table 8. Consistency evaluation of variant classifications

Sampling date of selected sequences	Sample size	Number of specific variants after undergoing reclassification by each nomenclature system [a]			Consistency for same batch of sequences of specific variants	
		Lineage call	Scorpio call	Nextclade	Lineage call versus Scorpio call	Lineage call versus Nextclade
2021-09-15	2322	2302	2302	2320	100.0% (2302/2302)	100.0% (2301/2301)
2021-06-15	1834	1757	1751	1761	100.0% (1751/1751)	99.8% (1753/1757)
2021-03-15	2088	1754	1706	1759	100.0% (1706/1706)	99.8 (1749/1753)
2020-12-15	2611	485	193	481	100.0% (193/193)	100.0% (484/484)
Total	8855	6298	5952	6321	100.0% (5952/5952)	99.9% (6287/6295)

[a] Specific variants include Alpha, Beta, Gamma, Delta, Lambda, Mu, Kappa, Epsilon, Theta, Iota, Eta, and EU1.

Supplementary Table 9. Categories of sequencing technologies

**Sequencing technologies**	**Sequencing platforms**
First generation sequencing	Sanger [a]
Second generation sequencing	Illumina®, BGI, MGI, BioelectronSeq, Ion Torrent
Third generation sequencing	Oxford Nanopore, PacBio Sequel, Clear Dx™ (based on Oxford Nanopore platform)

[a] Sanger sequencing technology is regarded as a type of sequencing platform here.

Supplementary Table 10. The cumulative official number of variants that used for calculating the extent of public availability of genomic data.

Country	Starting point (ISO week)	Ending point (ISO week)	Alpha [a]	Beta	Gamma	Delta
African Region						
Algeria	2020-01	2021-37	11	- [b]	-	25
South Africa	2020-01	2021-40	234	6959	-	9509
Democratic Republic of the Congo	2020-01	2021-31	16	32	-	228
Kenya	2020-01	2021-41	832	207	-	1674
Malawi	2020-01	2021-37	5	333	-	189
The Gambia	2020-01	2021-41	72	-	-	295
Senegal	2020-01	2021-31	219	3	-	264
Cameroon	2020-01	2021-29	43	17	-	14
Region of Americas						
Canada	2020-01	2021-40	268054	2416	21443	149823
Argentina	2021-01	2021-38	396	1	3373	561
Brazil	2021-02	2021-39	448	5	22380	13600
United States	2021-38	2021-41	4	3	17	129039
Peru	2020-01	2021-40	20	-	1843	2686
Chile	2020-01	2021-42	293	4	4328	5082
Ecuador	2020-01	2021-34	265	-	330	462
Panama	2020-01	2021-43	158	-	192	429
Eastern Mediterranean Region						
Jordan	2020-01	2021-11	3389	5	-	-
Libya	2020-01	2021-11	25	15	-	-
Iran	2020-01	2021-11	1792	-	-	-
Morocco	2020-01	2021-11	115	-	-	-
Tunisia	2020-01	2021-11	192	-	-	-
Iraq	2020-01	2021-11	236	-	-	-
Afghanistan	2020-01	2021-11	7	-	-	-
Saudi Arabia	2020-01	2021-11	10	-	-	-
European Region						
Switzerland	2020-01	2021-43	37584	521	256	31878
Denmark	2020-01	2021-43	68475	131	67	80620
Finland	2020-01	2021-40	8155	1460	8	10148
Austria	2020-01	2021-43	132033	1351	157	106976

Sweden	2020-01	2021-42	72578	2757	211	40100
Spain	2020-01	2021-41	23010	1386	1007	30383
Luxembourg	2020-01	2021-42	6971	1196	1216	4500
Netherlands	2020-01	2021-43	26497	442	383	29176
Ireland	2020-51	2021-41	16053	77	33	18691
Belgium	2021-20	2021-40	5316	78	728	27827
Cyprus	2020-01	2021-34	654	1	-	658
Estonia	2020-01	2021-40	5278	51	8	4861
France	2020-01	2021-40	10765	3605	1931	116308
Greece	2020-01	2021-39	16383	546	12	12019
Hungary	2020-01	2021-40	714	-	1	3371
Iceland	2020-01	2021-35	535	4	16	4947
Italy	2020-01	2021-40	28254	290	2878	31327
Latvia	2020-01	2021-40	3580	14	4	5157
Lithuania	2021-17	2021-38	8532	-	6	7825
Norway	2020-01	2021-40	39100	627	11	33545
Poland	2020-01	2021-40	19958	69	28	7481
Portugal	2020-01	2021-40	13771	112	193	10429
Romania	2020-01	2021-33	1501	8	20	1135
Slovakia	2020-01	2021-40	10182	29	-	7033
United Kingdom	2020-01	2021-42	277664	1104	290	1117204
Slovenia	2020-01	2021-42	9123	29	9	14959
South-East Asia Region						
India	2020-01	2021-40	4349	259	-	38519
Thailand	2020-01	2021-42	14629	690	-	26752
Indonesia	2021-01	2021-42	76	22	-	4732
Western Pacific Region						
Australia	2020-01	2021-40	568	100	8	20677
Philippines	2020-01	2021-33	3106	3562	-	5331
Malaysia	2020-01	2021-41	14	226	-	3522
New Zealand	2020-01	2021-41	178	33	8	1812
Japan	2020-01	2021-39	50240	117	137	73995
Cambodia	2020-01	2021-35	-	-	-	3713
China	2020-01	2021-20	102	46	3	38
South Korea	2020-01	2021-30	3045	148	20	4912

Laos	2020-01	2021-39	72	-	-	108

[a] From starting point to ending point of sampling time, the cumulative official number of Alpha variants in this country.

[b] No cases has been detected or the data was unavailable.

Note: A fixed three-week collection-to-report delay was used to extrapolate the date of specimen collection for Brazil, Canada, Peru, Chile, Ecuador, Finland, Spain, Belgium, Cyprus, Estonia, France, Greece, Hungary, Iceland, Italy, Latvia, Lithuania, Norway, Poland, Portugal, Romania, Slovakia, Australia, Philippines, Malaysia, Japan, China, Laos, India, Indonesia, all countries in Africa and Eastern Mediterranean Region.

Supplementary Table 11. An acknowledgement table for all originating laboratories contributed to generating sequences.
[table in Excel file]

Supplementary Table 12. An acknowledgement table for all submission laboratories contributed to sharing sequences.
[table in Excel file]

Reference

1. Rambaut, A. *et al.* A dynamic nomenclature proposal for SARS-CoV-2 lineages to assist genomic epidemiology. *Nat Microbiol* **5**, 1403-1407 (2020).
2. Konings, F. *et al.* SARS-CoV-2 Variants of Interest and Concern naming scheme conducive for global discourse. *Nat Microbiol* (2021).
3. World Health Organization. Scaling up genomic sequencing in Africa. (2021).
4. European Commission. Communication from the Commission to the European Parliament, the European Council and the Council - A united front to beat COVID-19. (2021).
5. Vavrek, D. *et al.* Genomic surveillance at scale is required to detect newly emerging strains at an early timepoint. *medRxiv*, 2021.01.12.21249613 (2021).
6. European Centre for Disease Prevention and Control. Guidance for representative and targeted genomic SARS-CoV-2 monitoring. (2021).
7. World Health Organization. Interim operational guidance on SARS-CoV-2 genomic surveillance in Africa: An updated guide. (2021).
8. Paul, P. *et al.* Genomic Surveillance for SARS-CoV-2 Variants Circulating in the United States, December 2020-May 2021. *MMWR Morb Mortal Wkly Rep* **70**, 846-850 (2021).
9. Molenkamp, R. *et al.* Supplementing SARS-CoV-2 genomic surveillance with PCR-based variant detection for real-time actionable information, the Netherlands, June to July 2021. *Euro Surveill* **26**(2021).
10. O'Toole, Á. *et al.* Assignment of epidemiological lineages in an emerging pandemic using the pangolin tool. *Virus Evol* **7**, veab064 (2021).
11. Slatko, B.E., Gardner, A.F. & Ausubel, F.M. Overview of Next-Generation Sequencing Technologies. *Curr Protoc Mol Biol* **122**, e59 (2018).
