# Supplementary material for: Global landscape of SARS-CoV-2 genomic surveillance and data sharing
Source: Nat Genet. 2022 Mar 28;54(4):499–507. doi: 10.1038/s41588-022-01033-y (PMC9005350; doi:10.1038/s41588-022-01033-y)
Supplement: Supplementary file 2 — Reporting Summary [file 41588_2022_1033_MOESM2_ESM.pdf]

nature portfolio

Corresponding author(s): Hongjie Yu
Last updated by author(s): Jan 11, 2022

Reporting Summary

Nature Portfolio wishes to improve the reproducibility of the work that we publish. This form provides structure for consistency and transparency in reporting. For further information on Nature Portfolio policies, see our Editorial Policies and the Editorial Policy Checklist.

Statistics

For all statistical analyses, confirm that the following items are present in the figure legend, table legend, main text, or Methods section.

n/a	Confirmed	
☐	☒	The exact sample size (n) for each experimental group/condition, given as a discrete number and unit of measurement
☒	☐	A statement on whether measurements were taken from distinct samples or whether the same sample was measured repeatedly
☐	☒	The statistical test(s) used AND whether they are one- or two-sided<br>*Only common tests should be described solely by name; describe more complex techniques in the Methods section.*
☒	☐	A description of all covariates tested
☒	☐	A description of any assumptions or corrections, such as tests of normality and adjustment for multiple comparisons
☐	☒	A full description of the statistical parameters including central tendency (e.g. means) or other basic estimates (e.g. regression coefficient) AND variation (e.g. standard deviation) or associated estimates of uncertainty (e.g. confidence intervals)
☐	☒	For null hypothesis testing, the test statistic (e.g. F, t, r) with confidence intervals, effect sizes, degrees of freedom and P value noted<br>*Give P values as exact values whenever suitable.*
☒	☐	For Bayesian analysis, information on the choice of priors and Markov chain Monte Carlo settings
☒	☐	For hierarchical and complex designs, identification of the appropriate level for tests and full reporting of outcomes
☒	☐	Estimates of effect sizes (e.g. Cohen's d, Pearson's r), indicating how they were calculated

Our web collection on statistics for biologists contains articles on many of the points above.

Software and code

Policy information about availability of computer code

Data collection	We collected data in a structured database on Microsoft Excel v.2019.
Data analysis	All data cleaning, statistical analyses, and visualizations were performed in R (version 4.0.2). The Pangolin (v3.1.16) and Nextstrain (Web 1.7.4) nomenclature systems were adopted to check the classification of variants. Data and code used in this study can be downloaded from GitHub at https://github.com/zychenfd/Global-landscape-of-SARS-CoV-2-variants.

For manuscripts utilizing custom algorithms or software that are central to the research but not yet described in published literature, software must be made available to editors and reviewers. We strongly encourage code deposition in a community repository (e.g. GitHub). See the Nature Portfolio guidelines for submitting code & software for further information.

Data

Policy information about availability of data

All manuscripts must include a data availability statement. This statement should provide the following information, where applicable:

- Accession codes, unique identifiers, or web links for publicly available datasets
- A description of any restrictions on data availability
- For clinical datasets or third party data, please ensure that the statement adheres to our policy

1. All the genomic data used in this analyses are available in 2019nCoVR (https://ngdc.cncb.ac.cn/ncov/release_genome) and GISAID (https://www.gisaid.org/). The accession numbers used in this study can be found in GitHub (https://github.com/zychenfd/Global-landscape-of-SARS-CoV-2-variants).
2. Officially aggregated dataset of SARS-CoV-2 variants have been deposited on GitHub (https://github.com/zychenfd/Global-landscape-of-SARS-CoV-2-variants).
3. The aggregated data on variants in the European Surveillance System are available in https://www.ecdc.europa.eu/en/publications-data/data-virus-variants-covid-19-eueea.

4. COVID-19 epidemic data are derived from WHO (https://covid19.who.int/info/).
5. Population data in 2020 are obtained from the United Nations (https://population.un.org/wpp/Download).
6. Socio-demographic index (SDI) in 2019 are available in IHME (http://ghdx.healthdata.org/record/ihme-data/gbd-2019-socio-demographic-index-sdi-1950-2019).
7. GDP per capita adjusted for purchasing power parity are available in The World Bank (https://data.worldbank.org/indicator/NY.GDP.MKTP.PP.CD).
8. Administrative boundaries were obtained from the database of Global Administrative Areas (GADM, https://gadm.org/).
9. Other data are presented in Supplementary Information.

Field-specific reporting

Please select the one below that is the best fit for your research. If you are not sure, read the appropriate sections before making your selection.

☒ Life sciences ☐ Behavioural & social sciences ☐ Ecological, evolutionary & environmental sciences

For a reference copy of the document with all sections, see nature.com/documents/nr-reporting-summary-flat.pdf

Life sciences study design

All studies must disclose on these points even when the disclosure is negative.

Sample size	No sample size calculation was performed. A total of 5.1 million SARS-CoV-2 sequence samples from public repositories was used, which were determined from a collection of genomic data in multiple repositories and initial deduplication in 2019nCoVR. We believe that the sample size was sufficient since it accounted for a relatively high proportion of confirmed cases.
Data exclusions	For sequences in public repositories, we removed those sequences of the non-human host, non-assignment of PANGO lineage, or incomplete information about date of collection (only year).
Replication	All data analyzed in this study are included in our main text, Supplementary Information, and GitHub. Code used in this study can be downloaded from GitHub at https://github.com/zychenfd/Global-landscape-of-SARS-CoV-2-variants.
Randomization	N/A. This is a descriptive study.
Blinding	N/A. This is a descriptive study.

Reporting for specific materials, systems and methods

We require information from authors about some types of materials, experimental systems and methods used in many studies. Here, indicate whether each material, system or method listed is relevant to your study. If you are not sure if a list item applies to your research, read the appropriate section before selecting a response.

Materials & experimental systems

n/a	Involved in the study	
☒	☐	Antibodies
☒	☐	Eukaryotic cell lines
☒	☐	Palaeontology and archaeology
☒	☐	Animals and other organisms
☒	☐	Human research participants
☒	☐	Clinical data
☒	☐	Dual use research of concern

Methods

n/a	Involved in the study	
☒	☐	ChIP-seq
☒	☐	Flow cytometry
☒	☐	MRI-based neuroimaging
