# Supplementary material for: Global landscape of SARS-CoV-2 genomic surveillance and data sharing
Source: Nat Genet. 2022 Mar 28;54(4):499–507. doi: 10.1038/s41588-022-01033-y (PMC9005350; doi:10.1038/s41588-022-01033-y)
Supplement: Supplementary file 3 — Peer Review Information [file 41588_2022_1033_MOESM3_ESM.pdf]

Peer Review Information

Journal: Nature Genetics
Manuscript Title: Global landscape of SARS-CoV-2 genomic surveillance and data sharing
Corresponding author name(s): Dr Hongjie Yu

Reviewer Comments & Decisions:

Decision Letter, initial version:

2nd Nov 2021

Dear Professor Yu,

Your Article, "Global landscape of SARS-CoV-2 genomic surveillance, public availability extent of genomic data, and epidemic shaped by variants" has now been seen by 3 referees. You will see from their comments below that while they find your work of interest, some important points are raised. We are interested in the possibility of publishing your study in Nature Genetics, but would like to consider your response to these concerns in the form of a revised manuscript before we make a final decision on publication.

To guide the scope of the revisions, the editors discuss the referee reports in detail within the team, with a view to identifying key priorities that should be addressed in revision. In this case, all referees have provided constructive reviews aimed at strengthening the analyses and clarifying some methodologies, and we particularly ask that you address their specific comments with appropriate revisions. We hope that you will find the prioritized set of referee points to be useful when revising your study.

We therefore invite you to revise your manuscript taking into account all reviewer and editor comments. Please highlight all changes in the manuscript text file. At this stage we will need you to upload a copy of the manuscript in MS Word .docx or similar editable format.

We are committed to providing a fair and constructive peer-review process. Do not hesitate to contact us if there are specific requests from the reviewers that you believe are technically impossible or unlikely to yield a meaningful outcome.

When revising your manuscript:

*1) Include a “Response to referees” document detailing, point-by-point, how you addressed each referee comment. If no action was taken to address a point, you must provide a compelling argument. This response will be sent back to the referees along with the revised manuscript.

*2) If you have not done so already please begin to revise your manuscript so that it conforms to our Article format instructions, available
here.
Refer also to any guidelines provided in this letter.

*3) Include a revised version of any required Reporting Summary:
https://www.nature.com/documents/nr-reporting-summary.pdf
It will be available to referees (and, potentially, statisticians) to aid in their evaluation if the manuscript goes back for peer review.
A revised checklist is essential for re-review of the paper.

Please be aware of our guidelines on digital image standards.

Please use the link below to submit your revised manuscript and related files:

[REDACTED]

Note: This URL links to your confidential home page and associated information about manuscripts you may have submitted, or that you are reviewing for us. If you wish to forward this email to co-authors, please delete the link to your homepage.

We hope to receive your revised manuscript within eight to twelve weeks. If you cannot send it within this time, please let us know.

Please do not hesitate to contact me if you have any questions or would like to discuss these revisions further.

Nature Genetics is committed to improving transparency in authorship. As part of our efforts in this direction, we are now requesting that all authors identified as ‘corresponding author’ on published papers create and link their Open Researcher and Contributor Identifier (ORCID) with their account on the Manuscript Tracking System (MTS), prior to acceptance. ORCID helps the scientific community achieve unambiguous attribution of all scholarly contributions. You can create and link your ORCID from the home page of the MTS by clicking on ‘Modify my Springer Nature account’. For more information please visit please visit www.springernature.com/orcid.

We look forward to seeing the revised manuscript and thank you for the opportunity to review your work.

Sincerely,

Wei

Wei Li, PhD
Senior Editor
Nature Genetics
New York, NY 10004, USA
www.nature.com/ng

Reviewers' Comments:

Reviewer #1:
Remarks to the Author:
The authors describe published SARS-CoV-2 sequences, and characterise national surveillance sequencing. Overall the analyses seem sensible, and the maps are nice visualisations. But the results presented here are readily available in various public websites (e.g. scroll down to the "insights" graphs on https://www.rockefellerfoundation.org/pandemicpreventioninstitute/). There's nothing wrong with publishing a paper capturing some of these, but it does limit the impact of the analysis.

For example, no effort is made to characterise any properties of the international sequence landscape other than major VOCs. What about technologies, genome coverage distributions, quality of released metadata, etc? These are aspects that in theory could be analysed in GISAID, but I think have had less attention.

One specific comment is on the definition of "routine surveillance" and "limited surveillance". The former is either 150 sequences or 10% per week, but I think this is both a low bar, and a very different level if, for example, there are 100K cases / week, then the latter is 10K, not 150. I guess the WHO defined these, but I think it limits the utility to just look at this low level. Better I think to actually compute the proportion sequenced per country, or make the bins a bit more informative (e.g. >10k / week, >1K / week, etc)

Finally, it's also difficult to tell if sequences are "surveillance", since sometimes hundreds of samples are collected from, say, a local hospital, which doesn't necessarily mean it captures the circulating diversity well.

Reviewer #2:
Remarks to the Author:
This article provides an overview of some important points of interest of the SARS-CoV-2 pandemic and public health strategies. They highlight the variability in genomic surveillance strategies in different countries, including sequencing capabilities and coverage of Variants of concern (VOC), and highlighting the occasionally low public availability of VOC sequences. They also assessed the prevalence of variants over time per regions and globally, showing the alpha variant being dominant at the beginning of 2021 to be now almost completely replaced by the delta variant.

This is the nice aggregation of data to illustrate the progression of variants reported in a comprehensive way, globally and by region, providing a useful insight for public health and genomic studies. It should be noted that this is submitted as a research article, but to me it seems the methods involved are more like that of a review or perspective. Nonetheless, the observations on genomics surveillance, sequence availability, and time and space distribution of VOC and VOI are of important use when interpreting and putting in perspective public health data.
While this paper presents a good summary of the current SARS-CoV genomic surveillance status, the view there do not seem to be completely novel, probably because of the nature of the approach. But It does provide a coherent comprehensive summary and trend of genomic surveillance of SARS-CoV-2, where public health researchers could benefit from it.

Some specific questions/comments:
1. Regarding the methodology and acquired data, this is a reanalyzing of previously published sequences and aggregation of publicly available data, presented in more readable ways.
It seems that the authors did not mention why they did not include some countries (e.g. the United States and Brazil) in fig 2 and table S6, while the information can be found (e.g. for the US public repository https://covid.cdc.gov/covid-data-tracker/#circulatingVariants).
Criteria used for the different classifications seem to be justified and encompassing the idea that the authors wanted to show.

2. Reviewing global data can be a challenge, as different countries obviously do not report their strategies and cases in a similar manner (or at all). Despite those concerns about the data review and collection, most of the references are from official health institutions, providing quality information and making some of this information relevant and of interest.
Have the authors considered local sources of information reporting in non-English local language? Would that increase the data coverage and accuracy (e.g. date of first report)?

3. In regards of "Dataset of sars-cov-2 sequences" the Sars-Cov-2 sequences extracted from public repositories and as previously reported (ref 42 of the manuscript, Song, S. et al. The Global Landscape of SARS-CoV-2 Genomes, Variants, and Haplotypes in 2019nCoVR. Genomics Proteomics Bioinformatics 18, 749-759 (2020)), does it mean that the SARS-CoV-2 variant typing data and associated meta information used in this paper were directly from 2019nCoVR repository? Or they re-processed in the same way as 2019nCoVR did, plus adding data from other sources? Clarification would be helpful.

4. Discussion Line 196 – 198: "However, our study observed a sharing extent of exceed 100% exists in some countries, likely due to delays in the official reporting of sequencing results, or the incomplete official reporting system." I suggest indicating which countries are the examples of exceeding 100% instead of only saying "some countries" because this exceeding 100% aspect seems have not been covered with details in Results section. In addition, I think the "sharing proportion" or "sharing extent" require more clear definition. Why exceed 100% could be caused by delay in reporting sequencing results? Isn't that the timestamp for the sequence data used should be their sample collection date? I am confused on this.

5. Have the authors conducted extra procedure to check the variant classification associated with the genome/gene sequences provided by the database? As far as I know the pango lineage and hence variant assignment in the public databases contain numerous errors and inconsistency.

6. Statistics seemed to have been used only once, to determine the difference in attached metadata (line 140). It does not seem needed anywhere else. The rest of the analysis is more qualitative, so no statistics seem needed.

7. In terms of clarity, notwithstanding some of the points mentioned above, this paper is generally clear in its conception, criteria, and development, and the authors mention important weaknesses to consider regarding this paper. Furthermore, the authors have referred to other important and relevant studies appropriately.

8. Figure S1. It seems that some countries, such as China and Singapore are not in the tables S1 and S2 indicating their data sources and the first reports of variants. How comprehensive are the summary of first report dates of variants? Explanation would be helpful. Also, it would be useful to directly put those first report date data on the table S2.

9. It would be very useful if the authors could upload their raw aggregated data to the Journal online supplementary section/some permanent online repository (GitHub) that were used to plot their graphs to promote data reusability in publications.

Reviewer #3:
Remarks to the Author:
In this article, Chen et al present a comprehensive meta-analysis of genomic surveillance data collection and sharing across the globe during the COVID19 pandemic. The authors show regional and temporal trends in genomic surveillance coverage, data sharing, metadata sharing, and detection and prevalence of four variants of concern (Alpha, Beta, Gamma, Delta) from March 2020 to September 2021. The study is built on an impressive program of data retrieval and curation, and research into local public health strategies around the world, including surveys of government websites, local news and, of course, viral sequence repositories such as GISAID. The study provides a simple, bird's eye view of how variants have shaped the course of the pandemic and seems like a useful resource, albeit one that will need to be updated continually as the pandemic progresses.

I don't have any major criticisms of the paper's methodologies or findings. In saying that, I must clarify that I am a genetics researcher and not an expert in public health, epidemiology, geography, demographics etc, which makes it difficult for me to criticise the technical aspects of this work. I am not completely convinced that Nature Genetics is the right home for a study of this nature (which doesn't have much to do with genetics), but this is for the editor to judge, not me.

While it is an interesting summary, I am also not completely convinced the article has told us anything new/impactful/surprising:

1. From the data of genomic surveillance coverage, the authors show that low-income countries generally have poor coverage. This seems obvious to me, although it is good to have some formal data quantifying this. Perhaps something useful could be learned from the exceptions: i.e. a careful examination of LMICs with high genomic surveillance coverage – what factors have allowed those countries to go against the trend? The ability to replicate those factors in other LMICs would be impactful.

2. The analysis of data sharing and metadata sharing shows there is a lot of variability between countries in the proportion of data sharing, but this is not clearly determined by income / region. I found it interesting that high income countries in Europe / Americas etc had high tendency for incomplete metadata sharing. It would be interesting to further explore the underlying reasons for this. For example, the authors could compare comparable European countries with good records and poor records of metadata sharing and identify factors (e.g. privacy regulations) that discriminate them. Again, understanding the factors that contribute to metadata sharing completeness would be impactful, as it might allow us to improve this issue.

3. The authors provide interesting visualisations to show the geographical and temporal dynamics of the spread of VOCs (alpha, beta, gamma, delta) throughout the pandemic. The visualisations are nice, but I cannot see how they offer any major advance on similar (but more interactive) visualisations available through Nextstrain (https://nextstrain.org/ncov/open/global), which is an excellent, public-facing resource. The authors should discuss the merits of their approach over Nextstrain (and other public resources) and explain what we can learn from their work that was not already known.

4. Another advantage of Nextstain is that it is continually updated. By contrast, this paper will be frozen in time. How do the authors plan to ensure that this work remains relevant? Could the analyses they have presented by embedded in a public-facing, interactive website that will be continually updated and maintained as the pandemic progresses? If so, how would this differ from Nexstrain?

I also found a handful of minor issues:

-typo row 85 "from publicly repositories"
-typo in row 196 "a sharing extent of exceed 100% exists in some countries"
-typo in row 198 "timely sharing of those enables to adequately contextualize local data"
-typo in row 211: "sharing of information in a secure and trusted channel in the context of protect patient anonymity and in accordance of local regulations"

Figure 3: it's difficult to distinguish the light grey in the middle of the time range spectrum from the white used to identify countries with no data available. I suggest making this more distinct. Furthermore, the 'data unavailable' category is grey in some figures and white in others - I suggest sticking with a single colour scheme across all figures.

Figure 5: the figure is a bit crowded, and I think it would benefit from some borders / separators between the different temporal groups/ranges.

Overall, I think this is an interesting piece of work and I enjoyed reading it. I have some doubts about the novelty and level of impact. However, if the editor believes it is suitable and sufficiently impactful for Nature Genetics, then I would not stand in the way.

Sincerely
Dr Ira Deveson

Author Rebuttal to Initial comments

Point-by-point response to reviewers' comments

Reviewer #1 (Remarks to the Author):

1. The authors describe published SARS-CoV-2 sequences, and characterise national surveillance sequencing. Overall the analyses seem sensible, and the maps are nice visualisations. But the results presented here are readily available in various public websites (e.g. scroll down to the "insights" graphs on https://www.rockefellerfoundation.org/pandemicpreventioninstitute/). There's nothing wrong with publishing a paper capturing some of these, but it does limit the impact of the analysis.

Response: We thank the reviewer for taking the time to evaluate our manuscript and for her/his positive summary and valuable comments. We are glad that he/she thinks our analyses are sensible and the maps are nice visualizations.

We acknowledge that our study is driven by the public data, and most of our analyses have been made possible by the sharing of genomic data and reporting of official data. However, we performed a more comprehensive search and did some new/impactful things in our study. For example, our findings indicate that many countries are not sharing genomic data, which we think it deserves attention especially when new Omicron variant have appeared. Besides, we provided a global network of SARS-CoV-2 genomic surveillance, which can inform which countries need to strengthen the genomic monitoring.

2. For example, no effort is made to characterise any properties of the international sequence landscape other than major VOCs. What about technologies, genome coverage distributions, quality of released metadata, etc? These are aspects that in theory could be analysed in GISAID, but I think have had less attention.

Response: We totally agree with this point that the properties of the international sequence landscape also deserve attention as it’s the cornerstone of our analysis. In addition to previous analyses for characterizing international sequences:
1. Describing the global sequences distribution and proportion of cases sequenced by WHO region (Fig. 1C-F, now changed into Fig. 2e-h);
2. Evaluating the completeness of some variables (e.g., geography, age, sex, date of collection) in released metadata (Table S5, now changed into Supplementary Table 6).
We have added the following analyses for better understanding the properties of the international sequences:

1) Describe the sequencing technologies.

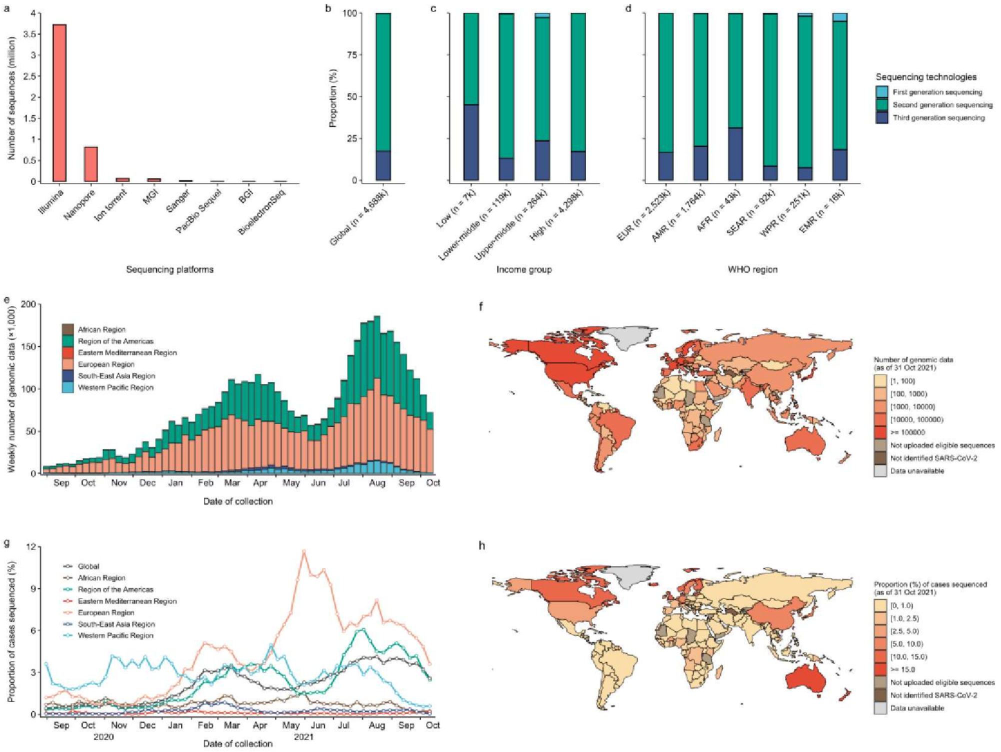

Figure 2. Sequencing technologies and distribution of global publicly deposited genomic data. a) Sequencing counts per sequencing platform. Sanger sequencing technology is regarded as a type of sequencing platform in this study. b-d) The proportions (%) of three types of sequencing technologies (e.g., first-generation sequencing, second-generation sequencing, and third-generation sequencing) used globally, by income group and WHO region. Here, we only present the data that were available with sequencing information (n = 4.69 million) in the GISAID as of Oct 31, 2021.

2) Calculate the turnaround time of SARS-CoV-2 sequences by income group, WHO region, and sampling time

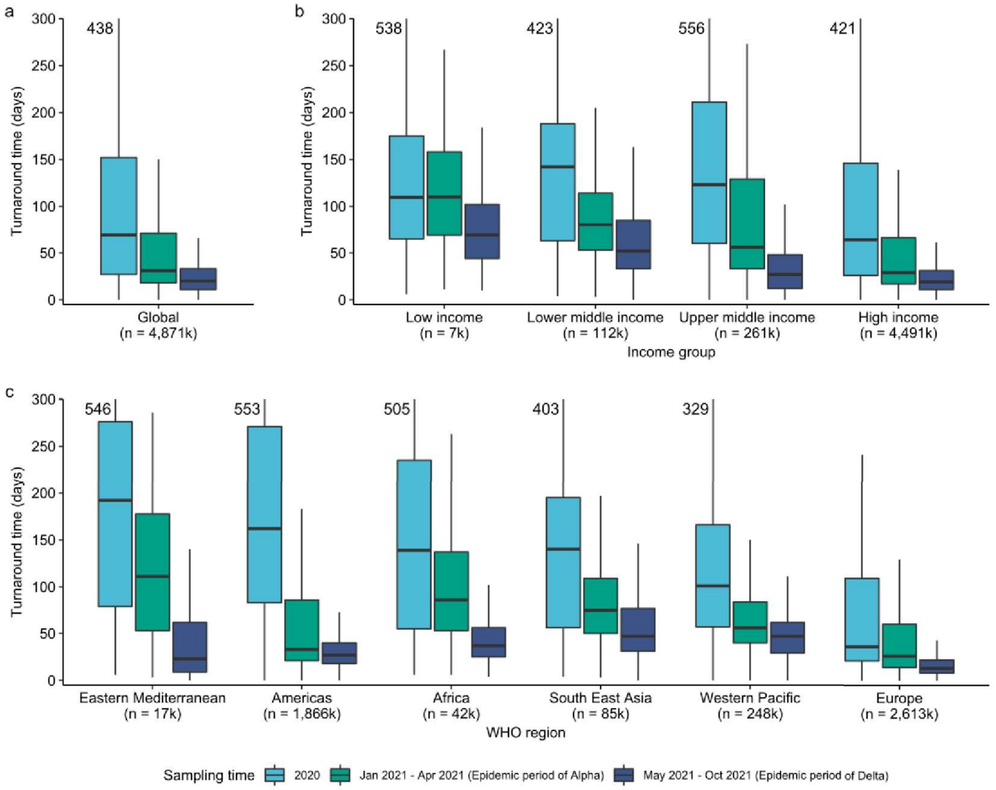

Extended Data Fig. 2 Distribution of turnaround time of SARS-CoV-2 sequences in different time periods. The turnaround time is defined as the time delay between specimen collection and data upload. The lower and upper hinges refer to the 25th and 75th percentiles, respectively; the lower and upper whiskers refer to the smallest values that are greater than or equal to the 1.5 interquartile range from the lower hinge and to the largest values that are further than the 1.5 interquartile range from the upper hinge, respectively. The centre line of each boxplot refers to the median value. Outlier points are not shown. We cut off the figure at a y-axis position of 300, and the values of the upper whisker that are beyond 300 are shown next to the bars.

3) Evaluate completeness of more variables in released metadata and develop a scoring system.

Based on previous Table S5 (now changed into Supplementary Table 6), we have added three variables (sample strategy, specimen source, sequencing method) for completeness evaluation, and analyzed the metadata completeness in different income groups. Besides, we have developed a scoring system to assess the metadata quality of each country based on the metadata completeness of ten vital variables (the weight of each variable is one point, and the total scores are 10 points) (Extended Data Fig. 5).

Supplementary Table 6. Completeness analysis of metadata collected from GISAID dataset by region and income groups.

	Total (N)	National geography [a]	Subnational geography [a]	Sample strategy	Specimen source	Sequencing method	Date of collection	Age	Sex	Patient status [b]	Vaccinated status [c]	Lineage
Global												
Total	4736168	100.0%	94.3%	16.1%	33.0%	99.0%	97.1%	37.1%	37.0%	3.6%	0.3%	97.9%
WHO region												
AFR	44004	100.0%	92.6%	28.3%	56.6%	98.6%	97.6%	87.3%	89.7%	14.1%	0.3%	96.5%
AMR	1781787	100.0%	99.8%	35.0%	58.2%	99.0%	97.2%	56.9%	57.9%	2.8%	0.3%	98.8%
EMR	15848	100.0%	75.4%	7.7%	63.3%	99.1%	94.9%	62.7%	64.4%	18.9%	0.9%	93.7%
EUR	2548298	100.0%	92.8%	4.6%	16.5%	99.0%	97.2%	21.7%	20.5%	2.9%	0.2%	97.5%
SEAR	92183	100.0%	98.1%	1.7%	41.2%	99.6%	91.5%	79.7%	82.2%	8.7%	2.0%	95.8%
WPR	254048	100.0%	70.0%	3.0%	13.0%	98.6%	97.4%	27.0%	28.6%	11.1%	0.1%	97.8%
Income group [d]												
Low	6541	100.0%	80.5%	10.7%	63.0%	99.4%	92.8%	72.9%	74.7%	18.4%	0.0%	98.5%
Lower middle	119682	100.0%	94.2%	10.1%	48.2%	99.6%	92.8%	82.1%	82.6%	19.4%	1.5%	96.2%
Upper middle	266268	100.0%	73.7%	5.5%	54.5%	99.1%	98.3%	57.9%	61.2%	20.8%	0.8%	96.3%
High	4343677	100.0%	95.5%	16.9%	31.2%	99.0%	97.1%	34.5%	34.2%	2.0%	0.2%	98.1%

[a] The completeness of geographic information (national or subnational) reported in metadata file.

[b] The completeness of information about patient status, includes the symptomatic history, clinical severity or outcome, etc.

[c] The completeness of vaccinated status, includes whether vaccinated or not, vaccinated platform, vaccinated dose, or vaccinated time, etc.

[d] The newest categories of income group for each country are obtained from the World Bank.

Abbreviations: AFR, African Region; AMR, Region of Americas; EMR, Eastern Mediterranean Region; EUR, European Region; SEAR, South-East Asia Region; WPR, Western Pacific Region. The data used is as of October 31, 2021.

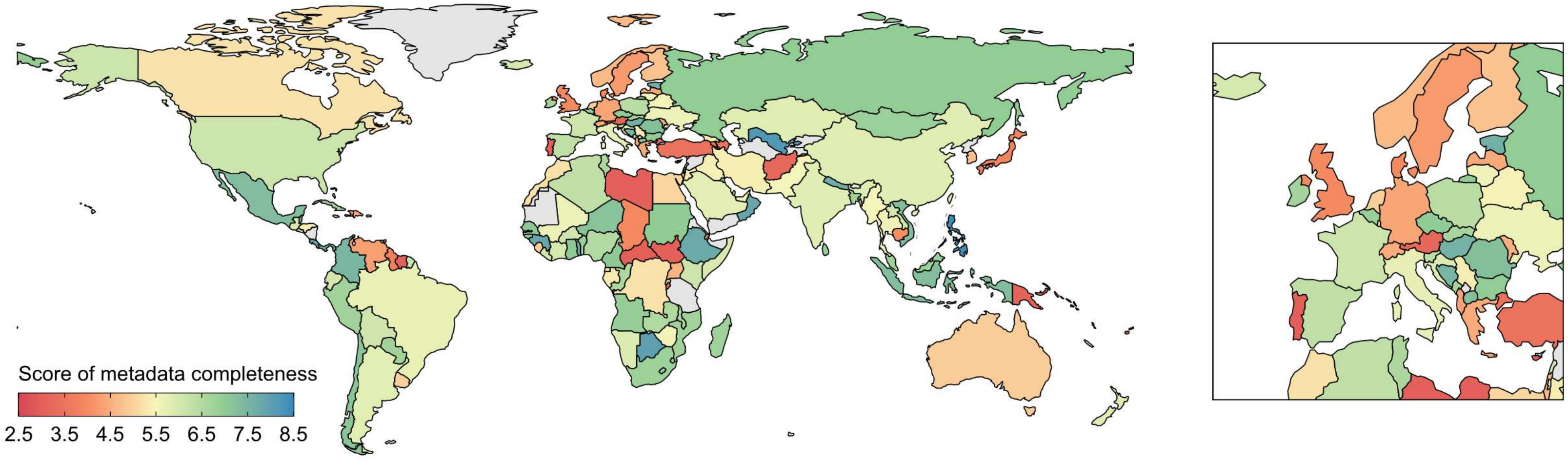

Extended Data Fig. 5 Total scores of metadata completeness. We developed a scoring system to assess the metadata quality of each country based on the metadata completeness of ten key variables, including subnational information, sample strategy, specimen source, sequencing technology, date of collection, sex, age, patient status, vaccinated status, and lineage (the weight of each variable is one point, and the total scores are 10 points). The right panel shows the expanded European region.

4) Extended analyses for two Variants of Interests (Lambda, Mu).

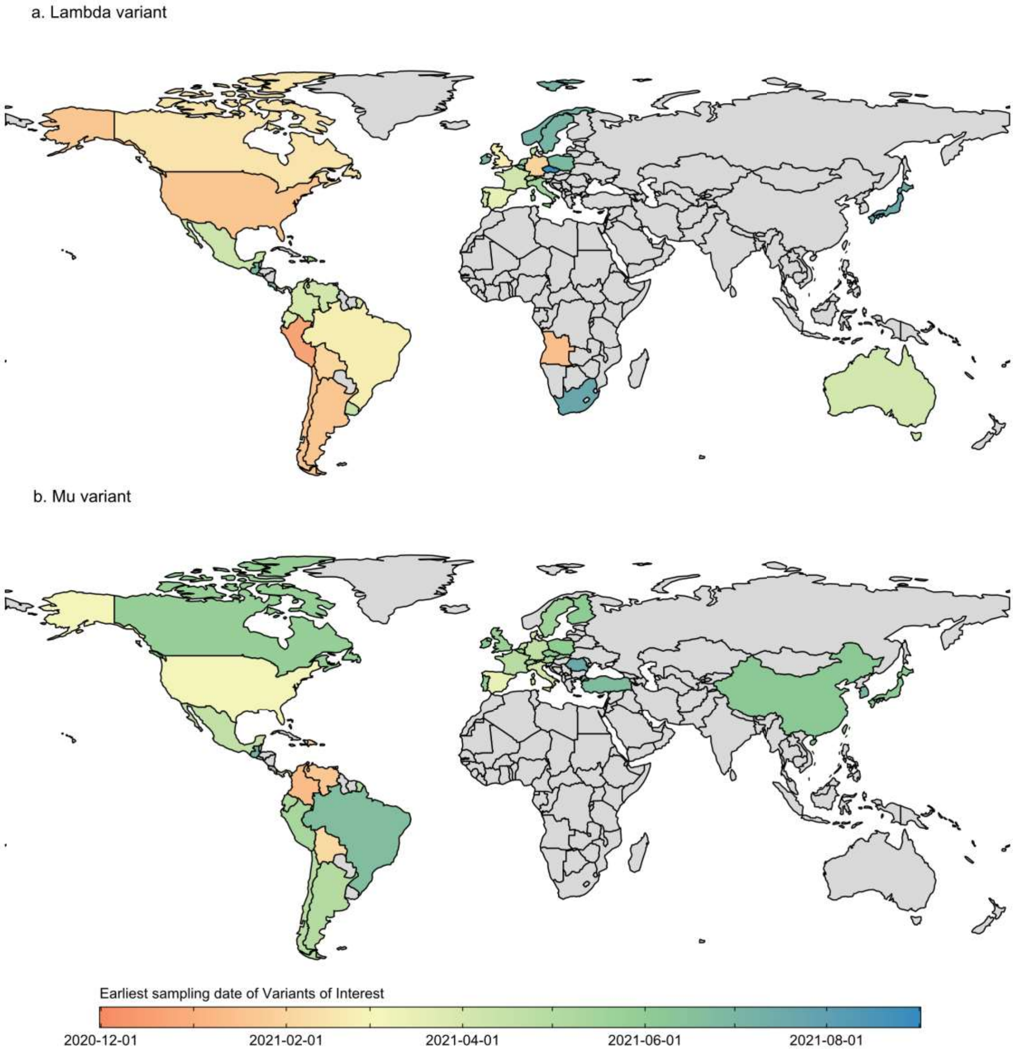

Extended Data Fig. 6 The earliest identification of the Lambda and Mu variants in each country. The grey areas represent countries with unreported variants or unavailable data.

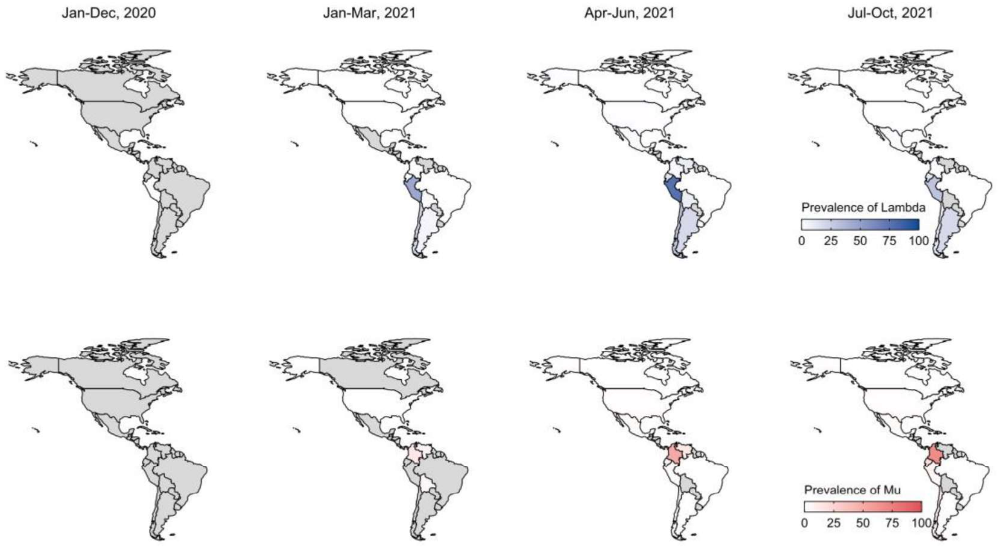

Extended Data Fig. 7 The prevalence and temporal dynamics of the Lambda and Mu variants in the Region of Americas. The countries that deposited more than 10 sequences in each period are included. Since the Lambda and Mu variants are circulating less widely in other regions, only the Region of Americas is presented in the map.

3. One specific comment is on the definition of "routine surveillance" and "limited surveillance". The former is either 150 sequences or 10% per week, but I think this is both a low bar, and a very different level if, for example, there are 100K cases / week, then the latter is 10K, not 150. I guess the WHO defined these, but I think it limits the utility to just look at this low level. Better I think to actually compute the proportion sequenced per country, or make the bins a bit more informative (e.g. >10k / week, >1K / week, etc).

Response: Thanks very much for pointing out this issue. we have thoroughly revised the definitions of different genomic surveillance strategies (Supplementary Table 3) and collected more data to fit this scheme per the reviewer's suggestion. In addition, we moved this table from Supplementary Information to the Extended Data.

Extended Data Table 1. Definitions of different genomic surveillance strategies.

<table>
<tr><th rowspan="3">Genomic surveillance strategy</th><th colspan="3">Definition</th></tr>
<tr><th rowspan="2">Strategy</th><th colspan="2">Sequencing volume (meet one of them)</th></tr>
<tr><th>Proportion</th><th>Smallest sequenced number per week [a]</th></tr>
<tr><td>High level of routine genomic surveillance</td><td rowspan="3">Regularly (per month or per week) collect nationwide samples to implement genomic sequencing</td><td>At least 5.0% of positive samples sequenced</td><td>A certain number of positive specimens sequenced that enables entity to detect a new variant at a prevalence of 1.0% tailored for this country with a specific range of number of new cases per week (e.g., 501-1000, or 1001-2500) based on a guideline published by European CDC</td></tr>
<tr><td>Moderate level of routine genomic surveillance</td><td>2.5-5.0% of positive samples sequenced</td><td>A certain number of positive specimens sequenced that enables entity to detect a new variant at a prevalence of 2.5% tailored for this country with a specific range of number of new cases per week (e.g., 501-1000, or 1001-2500) based on a guideline published by European CDC</td></tr>
<tr><td>Low level of routine genomic surveillance</td><td>1.0-2.5% of positive samples sequenced</td><td>A certain number of positive specimens sequenced that enables entity to detect a new variant at a prevalence of 5.0% tailored for this country with a specific range of number of new cases per week (e.g., 501-1000, or 1001-2500) based on a guideline published by European CDC</td></tr>
<tr><td>Limited genomic surveillance</td><td colspan="3">Conduct genomic sequencing, but with limited sequence capacity</td></tr>
</table>

[a] Refers to *Guidance for representative and targeted genomic SARS-CoV-2 monitoring* published by ECDC.

In this version, we classified the genomic surveillance strategy into four categories: 1) high level of routine genomic surveillance, 2) moderate level of routine genomic surveillance, 3) low level of routine genomic surveillance, and 4) limited genomic surveillance. The classification basis for regarding 5% of sequenced proportion as good routine genomic surveillance follows: 1) WHO recommendation for African countries [1]; 2) European Commission's recommendation for European countries [2]; 3) one modelling paper demonstrating that sequencing 5% of positive specimens allows the detection of emerging variants at a prevalence level of 0.1% to 1.0% [3]. In addition, we considered that countries with high burdens of newly confirmed cases probably struggle to achieve 5% sequencing proportions despite sequencing tens of thousands of positive specimens per week. Therefore, we also adopted an alternative definition for a high level routine genomic surveillance, namely, it is acceptable if the sequenced sample sizes are sufficient to detect a new variant at a prevalence of 1.0% that is tailored for locations that have a specific range of case numbers per week. The sample size was recommended by the ECDC guideline[4], as shown below (Table R1). For example, if a country has 70,000 cases per week (range: 50,001-100,000) and needs to detect a new variant at a prevalence of 1.0%, the required sample size for classification as a high level of routine genomic surveillance is 1,500 per week. That's to say, if this country sequenced more than 1,500 specimens per week, it will be regarded as high level of routine genomic surveillance; if sequenced more than 597 specimens per week, it will be regarded as moderate level of routine genomic surveillance; if sequenced more than 292 specimens per week, it will be regarded as low level of routine genomic surveillance; if sequenced less than 292 specimens per week, it will be regarded as limited genomic surveillance. The classifications of other three categories were similar, which were presented in Extended Data 1.

Table R1. Sample size needed to estimate the 95% confidence interval for the proportion of a certain circulating variant when its proportion reaches 1%, 2.5% or 5%.

Number of positive SARS-CoV-2 cases	Sample size based on the minimum prevalence of a variant to be detected		
	**1%**	**2.5%**	**5%**
> 100 000	1522	600	292
50001−100000	1500	597	292
25001−50000	1478	593	291
10001−25000	1435	586	289
5001−10000	1321	567	284
2501−5000	1167	536	276
1001−2500	947	484	262
501-1000	604	375	227
<500	337	273	185

This whole table was derived from *Guidance for representative and targeted genomic SARS-CoV-2 monitoring* published by ECDC. [4]

We have also clarified this in **Methods** (Line: 358-367) and **Supplementary Notes** (Page: 3):

> **Line 384-391**: To characterize the global landscape of SARS-CoV-2 genomic surveillance, we classified the surveillance strategy of each country into four categories: 1) high level of routine genomic surveillance; 2) moderate level of routine genomic surveillance; 3) low level of routine genomic surveillance; and 4) limited genomic surveillance. High/Moderate/Low level of routine genomic surveillance was defined as one entity regularly (per month or per week) collects nationwide samples to implement genomic sequencing, coupled with at least 5%/2.5%/1.0% of all positive samples sequenced [1-3], or a certain number of positive specimens sequenced that enables entity to detect a new variant at a prevalence of 1.0%/2.5%/5.0% tailored for this country with a specific range of number of new cases per week (e.g., 501-1000, or 1001-2500) based on a guideline published by European CDC[4] (Supplementary Notes, Extended Data Table 1).

4. Finally, it's also difficult to tell if sequences are "surveillance", since sometimes hundreds of samples are collected from, say, a local hospital, which doesn't necessarily mean it captures the circulating diversity well.

Response: To figure out this issue, we have tried to extract the information about sample strategy/source of nearly 5 million sequences deposited in GISAID, in which only 758k sequences have such information. After our grouping for those various types of sampling strategies, we found that 692k sequences appear to have been from routine surveillance (e.g., baseline surveillance, random sample, etc.), while 66k from targeted sampling (e.g., for imported travelers, breakthrough cases, cases in a local hospital, etc.). Although most sequences (91.3%) are from "surveillance" based on the limited analyses for those 758k sequences, but more than 80.0% sequences lack for the above information. For those reasons, it' s hard to tell if each sequence is obtained from surveillance network. However, given the nature of uniqueness of such public genomic data, we must recognize its value in accelerating and enhancing global scientific research.

We have added sentences to discuss this bias, and we also called for the standardizing of metadata to achieve this goal:

Line 255-256: The reliability of genomic data as a tool to capture local diversity of variant evolution and spread is dependent on the extent of available metadata from surveillance networks.

Line 298-300: After the new VOC emerging, more work is needed to define the ideal sampling schemes for various purposes and rapidly sharing and standardizing these data in public repositories for racing against virus.

Reviewer #2 (Remarks to the Author):

This article provides an overview of some important points of interest of the SARS-CoV-2 pandemic and public health strategies. They highlight the variability in genomic surveillance strategies in different countries, including sequencing capabilities and coverage of Variants of concern (VOC), and highlighting the occasionally low public availability of VOC sequences. They also assessed the prevalence of variants over time per regions and globally, showing the alpha variant being dominant at the beginning of 2021 to be now almost completely replaced by the delta variant.

This is the nice aggregation of data to illustrate the progression of variants reported in a comprehensive way, globally and by region, providing a useful insight for public health and genomic studies. It should be noted that this is submitted as a research article, but to me it seems the methods involved are more like that of a review or perspective. Nonetheless, the observations on genomics surveillance, sequence availability, and time and space distribution of VOC and VOI are of important use when interpreting and putting in perspective public health data.

While this paper presents a good summary of the current SARS-CoV genomic surveillance status, the view there do not seem to be completely novel, probably because of the nature of the approach. But It does provide a coherent comprehensive summary and trend of genomic surveillance of SARS-CoV-2, where public health researchers could benefit from it.

Response: We would like to thank the reviewer for taking the time to evaluate our study; we are glad that he/she believes our study "is a nice aggregation of data to illustrate the progression of variants reported in a comprehensive way" and "provide a coherent comprehensive summary and trend of genomic surveillance of SARS-CoV-2, where public health researchers could benefit from it".

Some specific questions/comments:

1. Regarding the methodology and acquired data, this is a reanalyzing of previously published sequences and aggregation of publicly available data, presented in more readable ways.

It seems that the authors did not mention why they did not include some countries (e.g. the United States and Brazil) in fig 2 and table S6, while the information can be found (e.g. for the US public repository https://covid.cdc.gov/covid-data-tracker/#circulatingVariants).

Criteria used for the different classifications seem to be justified and encompassing the idea that the authors wanted to show.

Response: Thanks for your positive summary for the criteria we used. The CDC COVID data tracker only uses sequence data that come from CDC contract surveillance programs, so these numbers are dramatically lower than the actual number of sequences, as well as

lower than the sequences deposited in GISAID/GenBank. Also, there is no cumulative reporting (absolute number) in the United States, and only recent four-week results are available (https://covid.cdc.gov/covid-data-tracker/#variant-proportions). With regards to Brazil, many results from local sequencing laboratories in Brazil have not yet been notified to National Network of Public Health Laboratories, thus the official variant number was not complete (https://www.gov.br/saude/pt-br/media/pdf/2021/outubro/29/boletim_epidemiologico_covid_86-final-_29out.pdf). Based on that, the United States and Brazil were previously not included. In this revision, we have added those two countries to give a more comprehensive view.

We had added a description of how to include and exclude aggregated data in **Methods**:

> **Line 335-336**: We only included those countries with such aggregated data available in Supplementary Table 3.
>
> **Line 406-410**: Some countries (e.g., the United States, Brazil) officially reported aggregated number of variants, of which agencies, however, acknowledged that the officially aggregated data may be incomplete due to its difficulty to capture the nationwide data. We still included these countries to give a more comprehensive view (Supplementary Table 3).

2. Reviewing global data can be a challenge, as different countries obviously do not report their strategies and cases in a similar manner (or at all). Despite those concerns about the data review and collection, most of the references are from official health institutions, providing quality information and making some of this information relevant and of interest. Have the authors considered local sources of information reporting in non-English local language? Would that increase the data coverage and accuracy (e.g. date of first report)?

Response: Yes, we have tried our best to collect the information in various languages by using the digital translations, and we have previously considered local sources of information reporting in non-English local language, which therefore made our data sources more abundant.

However, we must acknowledge that there might be some omissions, which has been added one sentence in our "**Limitations**":

> **Line 283-284**: Also, our efforts to collect and process multi-language data are limited by the accuracy and ability of digital translations.

3. In regards of "Dataset of sars-cov-2 sequences" the Sars-Cov-2 sequences extracted from

public repositories and as previously reported (ref 42 of the manuscript, Song, S. et al. The Global Landscape of SARS-CoV-2 Genomes, Variants, and Haplotypes in 2019nCoVR. Genomics Proteomics Bioinformatics 18, 749-759 (2020)), does it mean that the SARS-CoV-2 variant typing data and associated meta information used in this paper were directly from 2019nCoVR repository? Or they re-processed in the same way as 2019nCoVR did, plus adding data from other sources? Clarification would be helpful.

Response: We apologize for the lack of clarity. We downloaded the raw metadata from 2019nCoVR repository, in which they have integrated multiple genomic datasets and finished initial de-duplication process. Then, we double checked the duplicates of sequences and did the remaining data cleaning.

We have clarified it in **Methods**:

> **Line 317-321**: SARS-CoV-2 metadata file were downloaded from an online coronavirus analysis platform (2019nCoVR) on November 3, 2021, where has initially merged and deduplicated sequences that deposited in GISAID, GenBank, National Genomics Data Center, National Microbiology Data Center, and China National GeneBank. SARS-CoV-2 sequences was downloaded from the above original public repositories.
>
> **Line 373-378**: We double checked the duplicates of sequences by targeting those with the same virus name, date of collection, and country of collection as the 2019nCoVR repository defined for duplicates; we also treated the same accession ID identified as duplicates. After undergoing de-duplication, we removed key-variable-incomplete (date of collection, country of sampling, and assigned lineage) metadata and performed re-assignment of thousands of Pango lineages into WHO-designated variants. Detailed cleaning process for data in public repositories is shown in the Supplementary Notes.

To give an overview about our study, we also added a flowchart about data collection and data analysis:

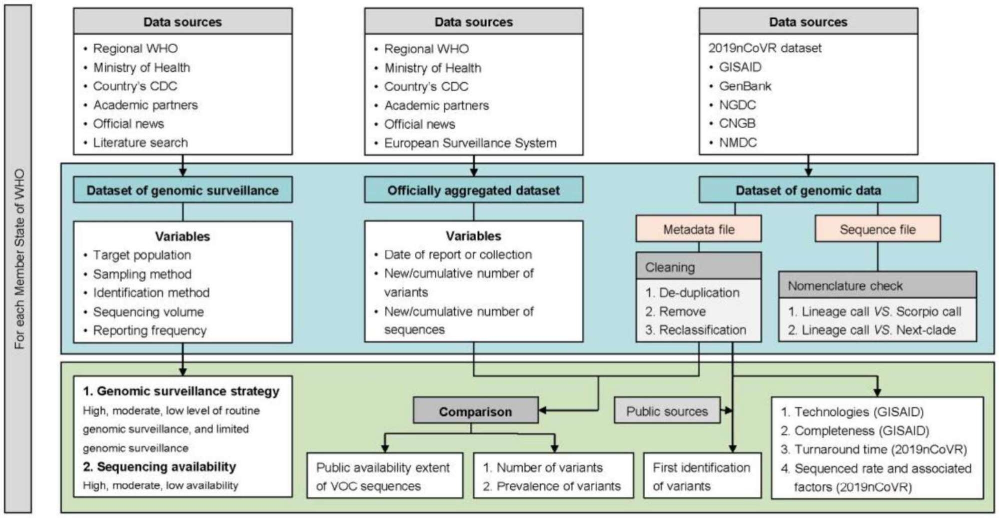

Extended Data Fig. 1 Overall flowchart of data collection and data analysis. NGDC, National Genomics Data Center; CNGB, China National GeneBank; NMDC, National Microbiology Data Center.

4. Discussion Line 196 – 198: "However, our study observed a sharing extent of exceed 100% exists in some countries, likely due to delays in the official reporting of sequencing results, or the incomplete official reporting system." I suggest indicating which countries are the examples of exceeding 100% instead of only saying "some countries" because this exceeding 100% aspect seems have not been covered with details in Results section. In addition, I think the "sharing proportion" or "sharing extent" require more clear definition. Why exceed 100% could be caused by delay in reporting sequencing results? Isn't that the timestamp for the sequence data used should be their sample collection date? I am confused on this.

Response: Thanks for your comments. To directly view which countries have a public availability extent of exceeding 100%, we have revised the legend of Fig 3 (previous Fig 2) by adding a unique-color legend of "> 100" extracting from legend of "> 75". In addition, we put the public availability extent of total VOC sequences in the main figure (Fig 3), whereas putting the extent of public availability across different VOC in Extend Data (Extended Data Fig. 4). We have added the information about which countries are the examples of exceeding 100% in **Figure 3 Legend**.

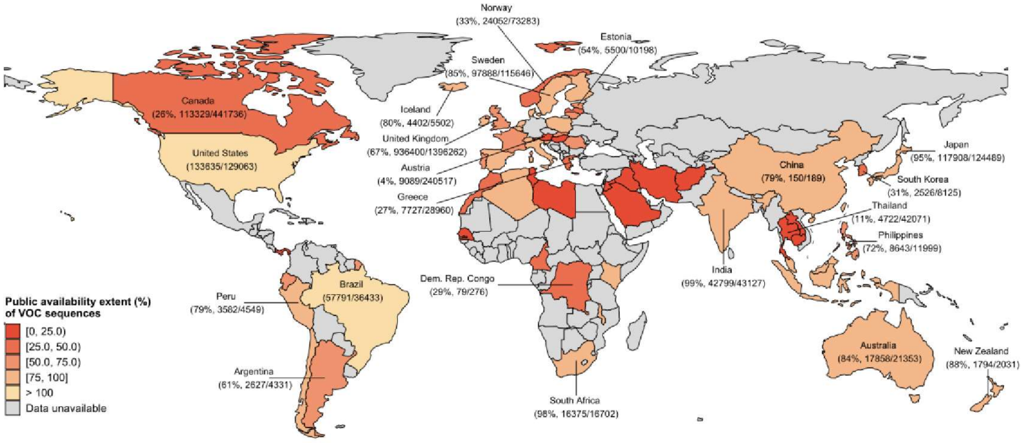

Fig 3. The public availability extent of VOC sequences to public repositories. In view of the availability of official data, the cumulative numbers of variants in different countries correspond to different time periods, with the detailed information contained in Supplementary Table 8. The variant data for China include those that have only been reported for mainland China. The officially reported number of alpha variants might contain those that were screened by PCR assays. The extent of public availability over 100% was observed in some countries (United States, Brazil), which was likely due to 1) inconsistent timestamps between the deposited genomic data and aggregated data (although we assumed a three-week collection-to-report time delay for Brazil, but this delay could be longer); 2) incomplete data aggregated in official reporting systems; or 3) the number of variants in genomic datasets that may be amplified by multiple sequences that were serially sampled from one patient at longitudinal timepoints. The sequences in public repositories with no collection dates for the specimens are not included. The Omicron variant was not included in this analysis, as most countries had not yet provided any officially reported data on the Omicron variant. The values beneath the country names indicate the numbers of cumulative variants during the same period: *variants in public repositories/official reported variants*.

We have revised "sharing proportion" or "sharing extent" into "public availability extent" in the draft to make the statement more precise and provided a more detailed definition in **Methods**, please see **Line 404-406**.

> **Line 404-406:** The public availability extent was defined as the ratio of the cumulative number of variants in public repositories to the official reported number of variants within the same period.

We apologize for the lack of clarity. The timestamps for the deposited genomic data we used are their sample collection date, but the timestamps of most of official data refers to reporting date. Although we assumed a three-week collection-to-report delay for such countries to align with the same scale of sample collection date, the time delay may be longer in some countries. For example, if officially aggregated data are reported more than 3 weeks after collection, the public availability extent might exceed 100%. In addition, the extent of public availability may also exceed 100% when the officially aggregated data is incomplete due to its difficulty to capture the nationwide data.

5. Have the authors conducted extra procedure to check the variant classification associated with the genome/gene sequences provided by the database? As far as I know the pango lineage and hence variant assignment in the public databases contain numerous errors and inconsistency.

Response: Thanks very much for reviewer raising the issue about accuracy of variant classification. To address this question, we consulted the technical personnel of 2019nCoVR repository, knowing that the variant classification in 2019nCoVR will be regularly updated based on the latest Pangolin tool. To check the consistency of latest “lineage call”, we did the following analyses:

First, we checked the consistency between “Lineage call” and “Scorpio call” for the same sequences. We conveniently selected some sequence samples that were collected at four timepoints (e.g., midpoints of December 2020, March 2021, June 2021, and September 2021), given the handling power of the online nomenclature systems. Then, we ran those sequences by using the Pangolin tool (https://pangolin.cog-uk.io/) to obtain the assigned “Lineage call” and “Scorpio call” for each sequence. We selected some specific variants (including Alpha, Beta, Gamma, Delta, Lambda, Mu, Kappa, Epsilon, Theta, Iota, Eta, and EU1) after undergoing classification by each nomenclature system to calculate the assignment consistencies. The sequences that were not assigned a classification were not included in the consistency analysis. For example, if a sequence was assigned to “none” by “Lineage call” but was assigned to “alpha-like” by “Scorpio call”, we removed this sequence to calculate the consistency. As a result, there was a high consistency (100.0%) between “lineage call” and “Scorpio call” (Supplementary Table 1).

Second, we also evaluated the degree of consistency between Pangolin and Nextstrain nomenclature systems for the same sequences downloaded as above. We ran these same sequences in the Pangolin tool (https://pangolin.cog-uk.io/) and Nextclade (https://clades.nextstrain.org/results) to assign lineage or clade classifications, respectively. Similarly, we selected some specific variants (including Alpha, Beta, Gamma, Delta, Lambda, Mu, Kappa, Epsilon, Theta, Iota, Eta, and EU1) after undergoing classification by

each nomenclature system to calculate assigned consistencies. Finally, we found that the degree of consistency reached 99.9% (Supplementary Table 1).

Although the nomenclature is sometimes changing at a rapid pace of the continuous updates and improvements of the Pangolin tools, incorrect lineage assignments still occasionally occur, especially when there is a certain proportion of ambiguous sites[9]. However, the genomic dataset (e.g., GISAID) assigns a lineage designation only for sequences that have less than 5% of ambiguous nucleotide sites in the coding regions[9]; therefore, the proportion of misallocations is expected to be very low.

Supplementary Table 1. Consistency evaluation of variant classifications

Sampling date of selected sequences	Sample size	Number of specific variants after undergoing reclassification by each nomenclature system [a]			Consistency for same batch of sequences of specific variants	
		Lineage call	Scorpio call	Nextclade	Lineage call versus Scorpio call	Lineage call versus Nextclade
2021-09-15	2322	2302	2302	2320	100.0% (2302/2302)	100.0% (2301/2301)
2021-06-15	1834	1757	1751	1761	100.0% (1751/1751)	99.8% (1753/1757)
2021-03-15	2088	1754	1706	1759	100.0% (1706/1706)	99.8 (1749/1753)
2020-12-15	2611	485	193	481	100.0% (193/193)	100.0% (484/484)
Total	8855	6298	5952	6321	100.0% (5952/5952)	99.9% (6287/6295)

[a] Specific variants include Alpha, Beta, Gamma, Delta, Lambda, Mu, Kappa, Epsilon, Theta, Iota, Eta, and EU1.

We have added some sentences to address this issue in **Methods** and put the above paragraphs in **Supplementary Notes**.

> **Line 378-383**: We also examined the potential misclassification of Pango lineage by comparing the consistency of "Lineage call" with "Scorpio call" and Nextstrain nomenclature systems through conveniently selecting about 9,000 sequences sampled in the midpoint of December 2020, March, June, and September 2021; we found the consistent degree reaches a level of 100.0% and 99.9%, respectively (Supplementary Table 1).

6. Statistics seemed to have been used only once, to determine the difference in attached metadata (line 140). It does not seem needed anywhere else. The rest of the analysis is more qualitative, so no statistics seem needed.

Response: Thanks for comments.

7. In terms of clarity, notwithstanding some of the points mentioned above, this paper is generally clear in its conception, criteria, and development, and the authors mention important weaknesses to consider regarding this paper. Furthermore, the authors have referred to other important and relevant studies appropriately.

Response: Thanks for your nice summary.

8. Figure S1. It seems that some countries, such as China and Singapore are not in the tables S1 and S2 indicating their data sources and the first reports of variants. How comprehensive are the summary of first report dates of variants? Explanation would be helpful. Also, it would be useful to directly put those first report date data on the table S2.

Response: For table S1 (now Supplementary Table 3), we only include data sources of some countries that have reported the aggregated data of variants, whereas those with no reporting will be not included. The aggregated data of China are obtained from China CDC that collected the number of variants detected in national genomic surveillance per week (unpublished work), and we have added China in Table S1 (now Supplementary Table 3); whereas the official reported number of variants is still unavailable in Singapore, thus not in the Table S1 (now Supplementary Table 3). As for table S2 (now Supplementary Table 4), if earliest sampling date of each VOC cannot be accessible in genomic datasets, we will search for other public sources for such information, which will display in the TableS2 (now Supplementary Table 4). The information about first identification of SARS-CoV-2 variants in China and Singapore was able to be obtained directly from genomic datasets, therefore not showing in TableS2 (now Supplementary Table 4). In addition, the data about first report date have be put on the Supplementary Table 4 (previous table S2) in the revision.

We have clarified how to include data into Supplementary Table 3 and 4 in **Methods**:

> **Line 335-336**: We only included those countries with such aggregated data available in Supplementary Table 3.
>
> **Line 426-427**: If a VOC was identified by WHO but no corresponding sequence in public repositories for one country, we used the date obtained from other sources (Supplementary Table 4).

9. It would be very useful if the authors could upload their raw aggregated data to the Journal online supplementary section/some permanent online repository (GitHub) that were

used to plot their graphs to promote data reusability in publications.

Response: Thanks for your advice. We will upload all data and codes to GitHub and will update in a regular frequency.

Reviewer #3 (Remarks to the Author):
In this article, Chen et al present a comprehensive meta-analysis of genomic surveillance data collection and sharing across the globe during the COVID19 pandemic. The authors show regional and temporal trends in genomic surveillance coverage, data sharing, metadata sharing, and detection and prevalence of four variants of concern (Alpha, Beta, Gamma, Delta) from March 2020 to September 2021. The study is built on an impressive program of data retrieval and curation, and research into local public health strategies around the world, including surveys of government websites, local news and, of course, viral sequence repositories such as GISAID. The study provides a simple, bird's eye view of how variants have shaped the course of the pandemic and seems like a useful resource, albeit one that will need to be updated continually as the pandemic progresses.

Response: We thank the reviewer for taking the time to evaluate our manuscript and for positive summary. We are glad that the reviewer believes that data retrieval, curation and research into local public health strategies around the world is impressive. We have updated all the data and analyses up to Oct 31, 2021 in the revised version.

I don't have any major criticisms of the paper’s methodologies or findings. In saying that, I must clarify that I am a genetics researcher and not an expert in public health, epidemiology, geography, demographics etc, which makes it difficult for me to criticise the technical aspects of this work. I am not completely convinced that Nature Genetics is the right home for a study of this nature (which doesn’t have much to do with genetics), but this is for the editor to judge, not me.

While it is an interesting summary, I am also not completely convinced the article has told us anything new/impactful/surprising:

Response: Thanks for your comments. We agree with your reasonable doubts about the novelty of our study. The main aims of our study are to provide a global mapping on how to conduct SARS-CoV-2 genomic surveillance globally, current degree of global sequencing, properties of uploaded genomic data, public availability extent of genomic data, as well as the trajectory of VOC identification and spread. The result at the macro-genetics level should be instructive to public health decision-making and global/regional cooperation. For example, first, our findings indicated that many countries are not sharing genomic data, which we think it has not been systematically addressed by other studies and should deserve attention. Second, we provided a global network of SARS-CoV-2 genomic surveillance, which can inform which countries need to strengthen the genomic monitoring and which countries need supportive networks for sequencing. Although the predefined purpose of our study partially limits our method novelty, but we thought the important issue deserves to be figured out especially when new Omicron variant have appeared.

1. From the data of genomic surveillance coverage, the authors show that low-income countries generally have poor coverage. This seems obvious to me, although it is good to have some formal data quantifying this. Perhaps something useful could be learned from the exceptions: i.e. a careful examination of LMICs with high genomic surveillance coverage – what factors have allowed those countries to go against the trend? The ability to replicate those factors in other LMICs would be impactful.

Response: The reviewer raised an important issue -- genomic surveillance disparities across different income groups. To quantify this, we have added a series of analyses in identifying the socioeconomic factors associated with sequencing percentage (Extended Data Fig. 3) and evaluating the difference of sequencing percentage by income groups (Supplementary Fig 1-2).

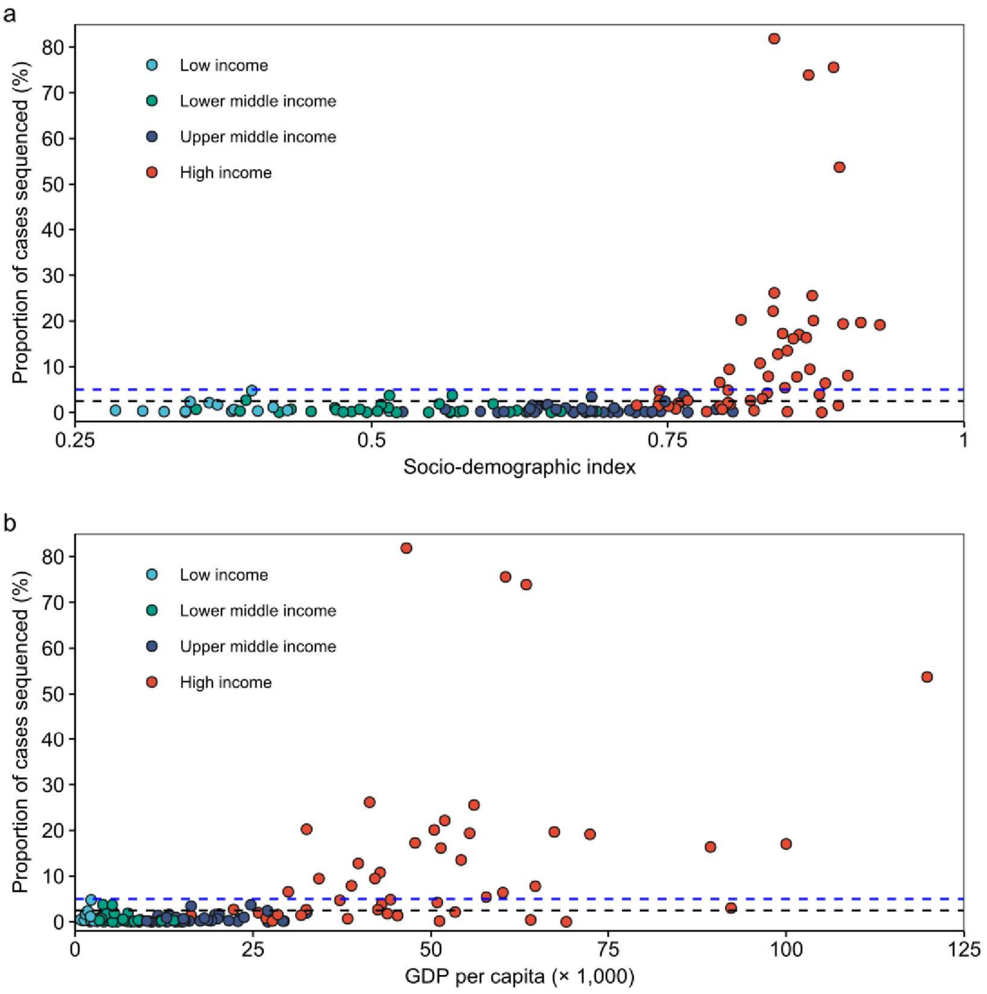

Extended Data Fig. 3 Proportions of cases sequenced in each country plotted against socioeconomic factors. a) Proportions of cases sequenced against the socio-demographic

index (SDI). The SDI can be divided into five categories: high, high-middle, middle, low-middle, and low. b) Proportions of cases sequenced against GDP per capita (unit: US dollars) that are adjusted for purchasing power parity. This analysis is restricted to the time period from May 1, 2021 to September 30, 2021, during which the delta variant began to dominate worldwide. Those countries that deposited fewer than 10 sequences in the genomic datasets from May 1, 2021 to September 30, 2021 were excluded. The blue and black horizontal dotted lines represent 5.0% and 2.5% of the sequenced percentage, respectively. Note: the sequenced percentage is a rough proxy that is due to the potential non-sharing of some genomic data and underreporting of cases.

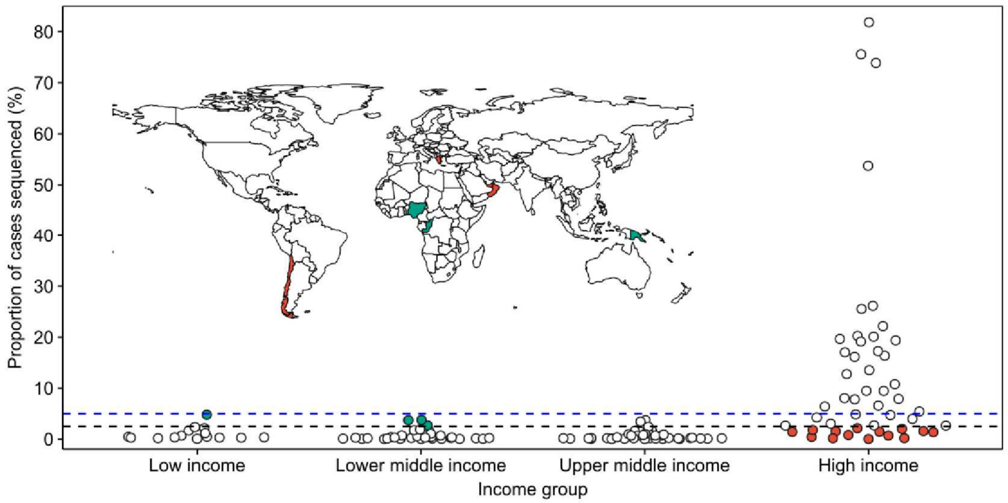

Supplementary Figure 1. Proportion of cases sequenced by income groups. Each dot represents one country that deposited at least 10 sequences from May 1, 2021 to September 30, 2021. The green dots refer to those low-income and lower middle-income countries with more than or equal to a 2.5% proportion of cases sequenced, which include Papua New Guinea, Nigeria, Congo, Rep., and The Gambia, while the red dots refer to those 14 high-income countries (e.g., Brunei Darussalam, Chile, Andorra, Greece, Israel, Malta, The Bahamas, Barbados, Trinidad and Tobago, Bahrain, Kuwait, Oman, United Arab Emirates, Seychelles) with less than a 2.5% proportion of infections sequenced. The blue and black horizontal dotted lines represent 5.0% and 2.5% of the sequenced percentages, respectively. Note: the sequenced percentage is a rough proxy due to the potential non-sharing of some genomic data and underreporting of confirmed cases.

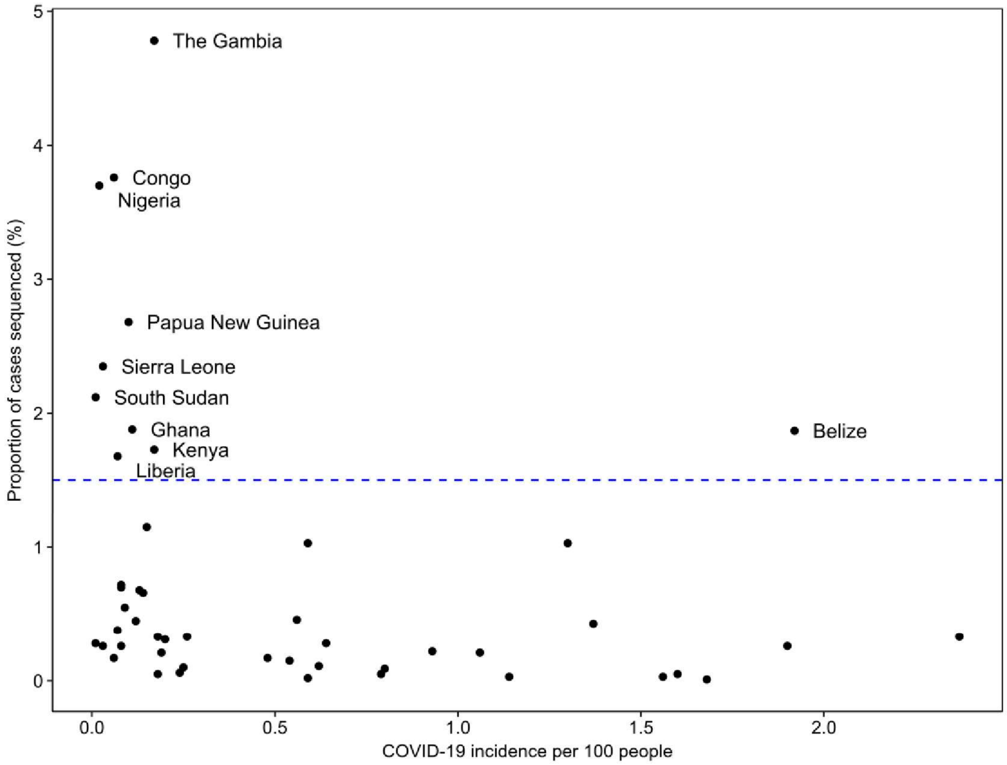

Supplementary Figure 2. Proportions of cases sequenced plotted against COVID-19 incidence levels per 100 people. Data from the low-income and lower middle-income countries are shown here. The blue horizontal dotted lines represent 1.5% of the sequenced percentage. We added the country name where the proportions of cases sequenced were more than 1.5%. Among the low- and lower middle-income countries, high sequencing percentages are mainly distributed in locations with low COVID-19 incidence rates.

We supplemented these sentences in **Results**:

> **Line 136-140**: We explored the relationship between socio-demographic index (SDI) and sequencing percentage and found that percentage was relatively constant at high-middle to low SDI levels with a sharp increase in coverage at high SDI (>0.805, Extended Data Fig. 3a). The relationship between GDP per capita and sequencing percentage followed the same general pattern with low percentage at low GDP values and a sharp increase in percentage (and variability) at higher levels (Extended Data Fig. 3b).

With regards to low-income and lower-middle income countries, we have tried to analysis the impact of COVID-19 incidence level on sequenced percentage, which indicated that relatively high sequenced percentage was mainly distributed in locations with low COVID-19 incidence rates. In addition, we found that the reference laboratories for SARS-CoV-2 genomic surveillance in Africa are partly located in Nigeria, Kenya, The

Gambia, and Ghana, which may be another potential reason why these countries perform well. Thus, we have summarized the potential factors to cause such a difference in **Discussion**:

> **Line 233-239**: Importantly, some low- and lower-middle income countries, such as The Gambia and Nigeria, were observed to have high proportion of cases sequenced in comparison to other countries in the same group. The precise factors underlying this discrepancy in sequencing capacity are unclear, but may include low COVID-19 incidence, accessibility to reference sequencing labs, as well as cooperative support from international groups and regional or local public health programs[27,28]. However, we note that sequenced proportion is a rough proxy for surveillance capacity, as this can be limited by a lack of sharing of genomic data and under-reporting of cases[29].

2. The analysis of data sharing and metadata sharing shows there is a lot of variability between countries in the proportion of data sharing, but this is not clearly determined by income / region. I found it interesting that high income countries in Europe / Americas etc had high tendency for incomplete metadata sharing. It would be interesting to further explore the underlying reasons for this. For example, the authors could compare comparable European countries with good records and poor records of metadata sharing and identify factors (e.g. privacy regulations) that discriminate them. Again, understanding the factors that contribute to metadata sharing completeness would be impactful, as it might allow us to improve this issue.

Response: Thanks for your comments. We have added some analyses in evaluating the sharing completeness of released metadata by income and region (Supplementary Table 6). We have also presented the completeness degree of metadata in all data-available countries (Supplementary Table 7), including the European countries with good records and poor records of metadata sharing.

Supplementary Table 6. Completeness analysis of metadata collected from GISAID dataset by region and income groups.

	Total (N)	National geography [a]	Subnational geography [a]	Sample strategy	Specimen source	Sequencing method	Date of collection	Age	Sex	Patient status [b]	Vaccinated status [c]	Lineage
Global												
Total	4736168	100.0%	94.3%	16.1%	33.0%	99.0%	97.1%	37.1%	37.0%	3.6%	0.3%	97.9%
WHO region												
AFR	44004	100.0%	92.6%	28.3%	56.6%	98.6%	97.6%	87.3%	89.7%	14.1%	0.3%	96.5%
AMR	1781787	100.0%	99.8%	35.0%	58.2%	99.0%	97.2%	56.9%	57.9%	2.8%	0.3%	98.8%
EMR	15848	100.0%	75.4%	7.7%	63.3%	99.1%	94.9%	62.7%	64.4%	18.9%	0.9%	93.7%
EUR	2548298	100.0%	92.8%	4.6%	16.5%	99.0%	97.2%	21.7%	20.5%	2.9%	0.2%	97.5%
SEAR	92183	100.0%	98.1%	1.7%	41.2%	99.6%	91.5%	79.7%	82.2%	8.7%	2.0%	95.8%
WPR	254048	100.0%	70.0%	3.0%	13.0%	98.6%	97.4%	27.0%	28.6%	11.1%	0.1%	97.8%
Income group [d]												
Low	6541	100.0%	80.5%	10.7%	63.0%	99.4%	92.8%	72.9%	74.7%	18.4%	0.0%	98.5%
Lower middle	119682	100.0%	94.2%	10.1%	48.2%	99.6%	92.8%	82.1%	82.6%	19.4%	1.5%	96.2%
Upper middle	266268	100.0%	73.7%	5.5%	54.5%	99.1%	98.3%	57.9%	61.2%	20.8%	0.8%	96.3%
High	4343677	100.0%	95.5%	16.9%	31.2%	99.0%	97.1%	34.5%	34.2%	2.0%	0.2%	98.1%

[a] The completeness of geographic information (national or subnational) reported in metadata file.

[b] The completeness of information about patient status, includes the symptomatic history, clinical severity or outcome, etc.

[c] The completeness of vaccinated status, includes whether vaccinated or not, vaccinated platform, vaccinated dose, or vaccinated time, etc.

[d] The newest categories of income group for each country are obtained from the World Bank.

Abbreviations: AFR, African Region; AMR, Region of Americas; EMR, Eastern Mediterranean Region; EUR, European Region; SEAR, South-East Asia Region; WPR, Western Pacific Region. The data used is as of October 31, 2021.

To compare the metadata sharing between comparable countries, we have developed a scoring system to assess the metadata quality of each country based on the metadata completeness degree of ten key variables (the weight of each variable is one point, and the total scores are 10 points) (Extend Data Fig. 5).

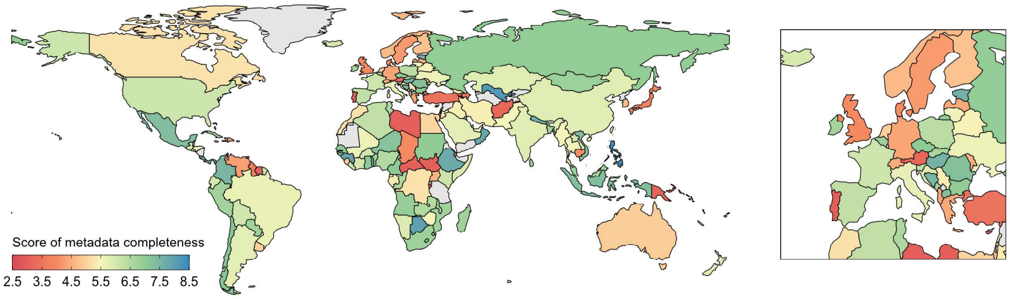

Extended Data Fig. 5 Total scores of metadata completeness. We developed a scoring system to assess the metadata quality of each country based on the metadata completeness of ten key variables, including subnational information, sample strategy, specimen source, sequencing technology, date of collection, sex, age, patient status, vaccinated status, and lineage. The right panel shows the expanded European region.

There are a lot of factors that might affect the sharing of genomic data and sharing completeness, in which precise reasons may be difficult to quantify. However, we tried to summarize the factors that might affect the public availability extent of genomic data in **Discussion**:

> **Line 243-253**: The discrepancies in data sharing were observed in each region, which confirmed that some countries are sequencing but are not uploading. Besides the initial concern about security of genomic data in a centralized repository, the fear of inequitable and incommensurate benefits from data sharing endeavors further dampens each agency's enthusiasm to upload data, especially in low-income countries[30]. An overarching challenge about how to protect the interests of data depositors to facilitate data sharing needs to be addressed, despite protection mechanisms (e.g., user identification, terms of access, data use agreements) provided by platforms such as GISAID[16]. Despite these challenges, it's still urgent to accelerate the speed (preferably real-time) and extent of submitting genomic data to publicly available databases for tracking of variants, since threats posted by the fast-spreading Omicron variant further reminds us the significance of data sharing[12].

We have also added sentences to discuss the factors that might affect the sharing completeness of metadata in **Discussion**:

Line 256-259: Different technical, economic, legal, and political barriers may impede the sharing of complete patient-level metadata[31], with impact across countries of all income levels, as illustrated by our finding of a frequent lack of demographic information shared by high-income countries.

3. The authors provide interesting visualisations to show the geographical and temporal dynamics of the spread of VOCs (alpha, beta, gamma, delta) throughout the pandemic. The visualisations are nice, but I cannot see how they offer any major advance on similar (but more interactive) visualisations available through Nextstrain (https://nextstrain.org/ncov/open/global), which is an excellent, public-facing resource. The authors should discuss the merits of their approach over Nextstrain (and other public resources) and explain what we can learn from their work that was not already known.

Response: Thanks for your comments. Undoubtedly, Nextstrain is a very useful interactive resource for tracking the phylogeny and spread of infectious pathogen. In terms of the geographical and temporal dynamics of the spread of VOCs, out study has the following differences: 1) we plotted the geographical and temporal dynamics of the spread of VOCs by employing a richer data source, which combined genomes from multiple genomic databases as well as the reported number from official aggregated dataset. However, the Nextstrain only subsamples about 4,000 sequences for each of the public builds. 2) The difference in sample size cause a slight difference in results. Citing an example of the African Region, our study showed that the proportion of Delta variant has reached a level of more than 90% in the early of July, whereas the proportion of that was 87% in Nextstrain. 3) We also presented the weekly counts of each variant, which was not done by Nextstrain. 4) The region categories we used was different, our study adopted the category of WHO region for showing, but the Nextstrain used another region category (Africa, Asia, Europe, North America, Oceania, South America).

In addition, the more prominent difference comes from the objective of our study, which aims to provide a global landscape of SARS-CoV-2 genomic surveillance and genomic data's sharing for informing actionable policy, both of which are not the focus of Nextstrain and other public resources.

We have added some sentences to discuss the difference between Nextstrain and our study:

Line 270-275: There are several web-based platforms that have provided up-to-date visualizations of SARS-CoV-2 genomic data and geographic distribution of variants, such as Nextstrain[38] and outbreak.info[39]. Tools such as these have played in invaluable part in dissemination and interpretation of SARS-CoV-2 genomic epidemiology data. However, our analyses extend beyond

the information contained in these tools. For example, the currently hosted regional Nextstrain pages for SARS-CoV-2 are, by necessity, subsampled from the 5 million sequences in GISAID to around 3,000 - 4,000 sequences.

4. Another advantage of Nextstain is that it is continually updated. By contrast, this paper will be frozen in time. How do the authors plan to ensure that this work remains relevant? Could the analyses they have presented by embedded in a public-facing, interactive website that will be continually updated and maintained as the pandemic progresses? If so, how would this differ from Nexstrain?

Response: It's a good suggestion. In this revision, we have updated our data and results up to Oct 31, 2021. All data and codes will be provided in GitHub and will update it in a regular frequency. The difference between Nextstrain and our study has been answered in last comment.

5. I also found a handful of minor issues:

-typo row 85 "from publicly repositories"
-typo in row 196 "a sharing extent of exceed 100% exists in some countries"
-typo in row 198 "timely sharing of those enables to adequately contextualize local data"
-typo in row 211: "sharing of information in a secure and trusted channel in the context of protect patient anonymity and in accordance of local regulations"

Response: Thanks for your typo correction. Done.

6. Figure 3: it's difficult to distinguish the light grey in the middle of the time range spectrum from the white used to identify countries with no data available. I suggest making this more distinct. Furthermore, the 'data unavailable' category is grey in some figures and white in others - I suggest sticking with a single colour scheme across all figures.

Response: Thanks for your advice, we have adjusted the colour scheme of Fig3 (now Fig 4) and aligned grey for 'data unavailable' category within all figures.

7. Figure 5: the figure is a bit crowded, and I think it would benefit from some borders / separators between the different temporal groups/ranges.

Response: Thanks for your advice, we have added more spaces between panels.

8. Overall, I think this is an interesting piece of work and I enjoyed reading it. I have some doubts about the novelty and level of impact. However, if the editor believes it is suitable

and sufficiently impactful for Nature Genetics, then I would not stand in the way.

Response: Thanks for your interests and comment.

To provide more insights about genome properties, we have added the following analyses in the **Extended Data**:

1) Describe the sequencing technologies.

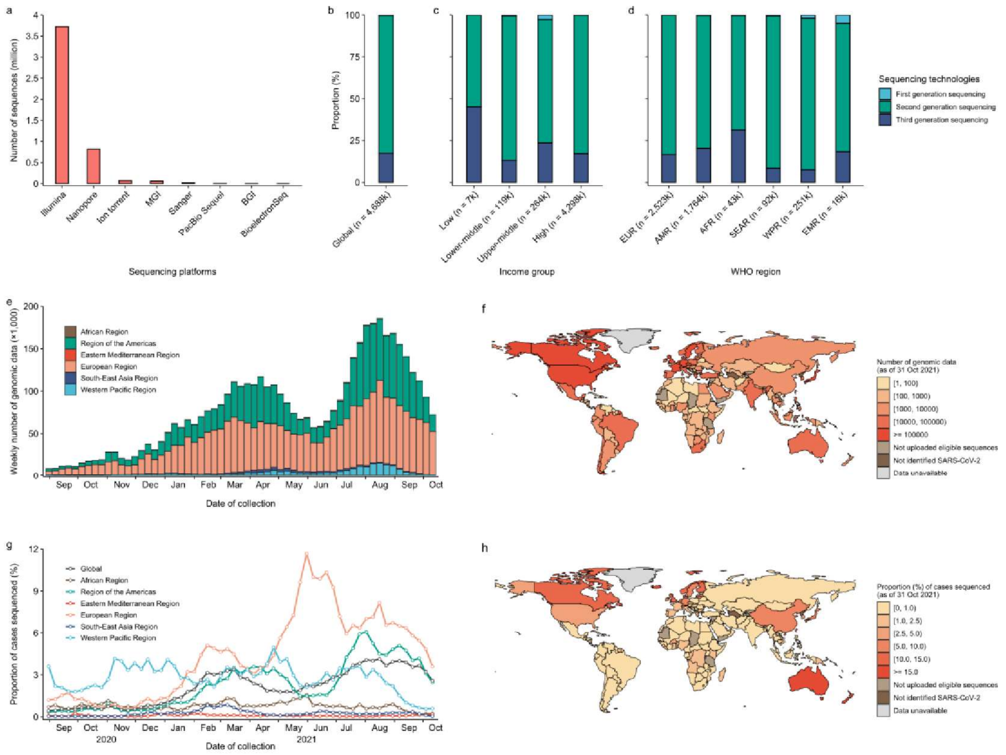

Figure 2. Sequencing technologies and distribution of global publicly deposited genomic data. a) Sequencing counts per sequencing platform. Sanger sequencing technology is regarded as a type of sequencing platform in this study. b-d) The proportions (%) of three types of sequencing technologies (e.g., first-generation sequencing, second-generation sequencing, and third-generation sequencing) used globally, by income group and WHO region. Here, we only present the data that were available with sequencing information (n = 4.69 million) in the GISAID as of Oct 31, 2021.

2) Calculate the turnaround time of SARS-CoV-2 sequences by income group, WHO region, and sampling time

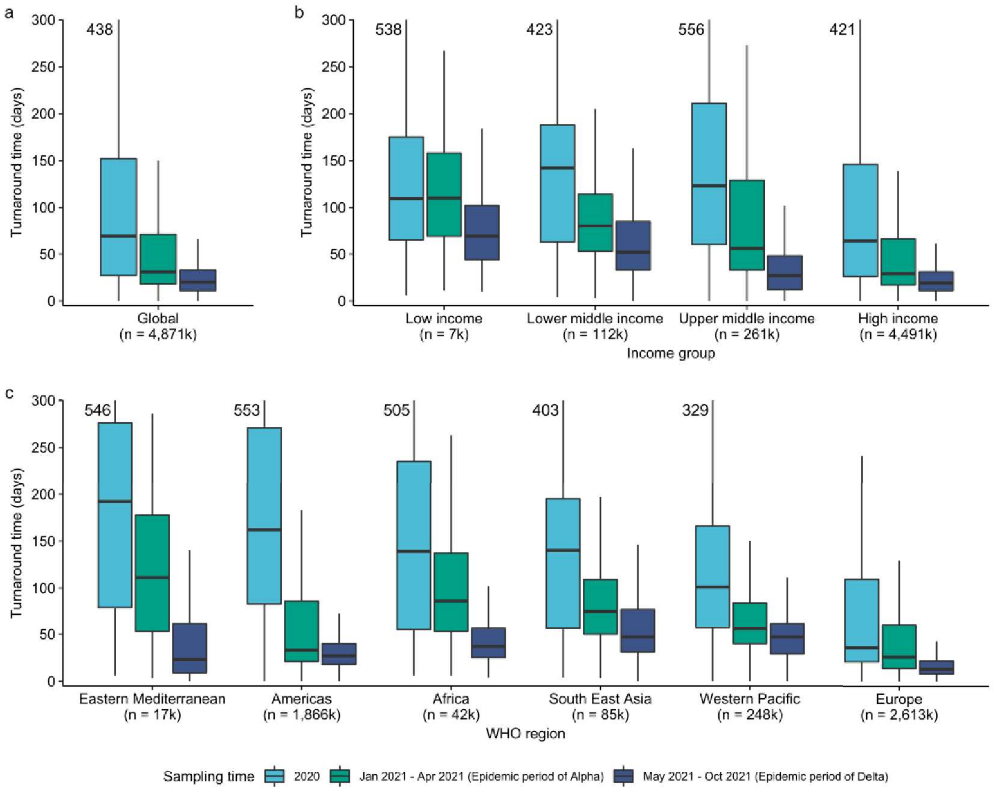

Extended Data Fig. 2 Distribution of turnaround time of SARS-CoV-2 sequences in different time periods. The turnaround time is defined as the time delay between specimen collection and data upload. The lower and upper hinges refer to the 25th and 75th percentiles, respectively; the lower and upper whiskers refer to the smallest values that are greater than or equal to the 1.5 interquartile range from the lower hinge and to the largest values that are further than the 1.5 interquartile range from the upper hinge, respectively. The centre line of each boxplot refers to the median value. Outlier points are not shown. We cut off the figure at a y-axis position of 300, and the values of the upper whisker that are beyond 300 are shown next to the bars.

Reference

1. World Health Organization. Scaling up genomic sequencing in Africa. (2021).
2. European Commission. Communication from the Commission to the European Parliament, the European Council and the Council - A united front to beat COVID-19. (2021).
3. Vavrek, D. *et al.* Genomic surveillance at scale is required to detect newly emerging strains at an early timepoint. *medRxiv*, 2021.01.12.21249613 (2021).
4. European Centre for Disease Prevention and Control. Guidance for representative and targeted genomic SARS-CoV-2 monitoring. (2021).
5. Schwalbe, N., Wahl, B., Song, J. & Lehtimaki, S. Data Sharing and Global Public Health: Defining What We Mean by Data. *Front Digit Health* **2**, 612339 (2020).
6. LoTempio, J. *et al.* We Can Do Better: Lessons Learned on Data Sharing in COVID-19 Pandemic Can Inform Future Outbreak Preparedness and Response. *Science & Diplomacy* **9**, 2.
7. O'Toole, Á. *et al.* Assignment of epidemiological lineages in an emerging pandemic using the pangolin tool. *Virus Evol* **7**, veab064 (2021).
8. Onwuamah, C.K. *et al.* SARS-CoV-2 sequencing collaboration in west Africa shows best practices. *Lancet Glob Health* **9**, e1499-e1500 (2021).
9. Otu, A., Agogo, E. & Ebenso, B. Africa needs more genome sequencing to tackle new variants of SARS-CoV-2. *Nat Med* **27**, 744-745 (2021).
10. Whittaker, C. *et al.* Under-reporting of deaths limits our understanding of true burden of covid-19. *Bmj* **375**, n2239 (2021).
11. Maxmen, A. Why some researchers oppose unrestricted sharing of coronavirus genome data. *Nature* **593**, 176-177 (2021).
12. Elbe, S. & Buckland-Merrett, G. Data, disease and diplomacy: GISAID's innovative contribution to global health. *Glob Chall* **1**, 33-46 (2017).
13. Hadfield, J. *et al.* Nextstrain: real-time tracking of pathogen evolution. *Bioinformatics* **34**, 4121-4123 (2018).
14. UK Health Security Agency. SARS-CoV-2 variants of concern and variants under investigation in England Technical briefing 27. (2021).

Decision Letter, first revision:

Our ref: NG-A58519R

16th Dec 2021

Dear Dr. Yu,

Thank you for submitting your revised manuscript "Global landscape of SARS-CoV-2 genomic surveillance, public availability extent of genomic data, and epidemic shaped by variants" (NG-A58519R). It has now been seen by the original referees and their comments are below. The reviewers find that the paper has improved in revision, and therefore we'll be happy in principle to publish it in Nature Genetics, pending minor revisions to satisfy the referees' final requests and to comply with our editorial and formatting guidelines.

If the current version of your manuscript is in a PDF format, please email us a copy of the file in an editable format (Microsoft Word or LaTex)-- we can not proceed with PDFs at this stage.

We are now performing detailed checks on your paper and will send you a checklist detailing our editorial and formatting requirements soon. Please do not upload the final materials and make any revisions until you receive this additional information from us.

Thank you again for your interest in Nature Genetics Please do not hesitate to contact me if you have any questions.

Sincerely,
Wei

Wei Li, PhD
Senior Editor
Nature Genetics
New York, NY 10004, USA
www.nature.com/ng

Reviewer #2 (Remarks to the Author):

The authors have addressed my comments in the revision. I don't have further comments except a minor one - fig. 3 the colour gradient may be better to be reversed, i.e. lighter-to-darker for lower-to-higher, so to be consistent with the light grey indicating data unavailability. There are some minor typos such as [) in the figure.

Reviewer #3 (Remarks to the Author):

I appreciate the additional work that the authors have completed in order to address the comments from all three reviewers. This has improved the manuscript in my opinion.

I am generally satisfied with the responses and I think several of the new figures are quite informative, eg Figure 2, Extended Data Fig. 5 and Supplementary Figures 1&2.

In their discussion of Supplementary Figures 1+2, the authors focus on the low- and lower-middle income countries with higher than average sequencing coverage (eg The Gambia and Nigeria). I think some attention is also warranted for the opposite group of outliers: those high-income countries with lower than average sequencing coverage (red dots in Supplementary Figure 1. What are the likely explanations for these outliers?

Besides that final point, I am happy with the updated version of the manuscript and I congratulate the authors on a nice piece of work overall.

Author Rebuttal, first revision:

Point-by-point response to reviewers' comments

Reviewer #2 (Remarks to the Author):

The authors have addressed my comments in the revision. I don't have further comments except a minor one - fig. 3 the colour gradient may be better to be reversed, i.e. lighter-to-darker for lower-to-higher, so to be consistent with the light grey indicating data unavailability. There are some minor typos such as [) in the figure.

Response: We thank the reviewer for taking the time to re-evaluate our study, also thank for your positive feedback on our revised manuscript. Following your suggestion, we have reversed the colour gradient for Fig 3 and Extended Data Fig. 4-5. We have also checked the typos of our manuscript.

Reviewer #3 (Remarks to the Author):

1. I appreciate the additional work that the authors have completed in order to address the comments from all three reviewers. This has improved the manuscript in my opinion.

I am generally satisfied with the responses and I think several of the new figures are quite informative, eg Figure 2, Extended Data Fig. 5 and Supplementary Figures 1&2.

Response: We thank the reviewer for taking the time to re-evaluate our study, and we are glad to hear that you are generally satisfied with our responses and happy with our revised manuscript.

2. In their discussion of Supplementary Figures 1+2, the authors focus on the low- and lower-middle income countries with higher than average sequencing coverage (eg The Gambia and Nigeria). I think some attention is also warranted for the opposite group of outliers: those high-income countries with lower than

average sequencing coverage (red dots in Supplementary Figure 1. What are the likely explanations for these outliers?

Response: Thanks for your advice. We have added the **Supplementary Fig 3** to plot the sequenced proportions against COVID-19 incidence levels in high-income countries. We have also added some sentences to discuss the underlying reasons leading to these outliers in high-income countries.

> **Line 240-244**: A country's income level is not the only factor affecting apparent viral sequencing capacity, as low sequencing proportions were also observed in high-income countries (e.g., United Arab Emirates, Kuwait) (Supplementary Figure 3). These apparent low sequencing proportions might be attributed to high COVID-19 incidence, poor genomic surveillance system, strict regulations governing biospecimens and data sharing, as well as differing norms on public data sharing[1].

3. Besides that final point, I am happy with the updated version of the manuscript and I congratulate the authors on a nice piece of work overall.

Response: Thanks.

Reference

1. Crawford, D.C. & Williams, S.M. Global variation in sequencing impedes SARS-CoV-2 surveillance. *PLoS Genet* **17**, e1009620 (2021).

Final Decision Letter:

In reply please quote: NG-AN58519R1 Yu

11th Feb 2022

Dear Dr. Yu,

I am delighted to say that your manuscript "Global landscape of SARS-CoV-2 genomic surveillance and data sharing" has been accepted for publication in an upcoming issue of Nature Genetics.

Over the next few weeks, your paper will be copyedited to ensure that it conforms to Nature Genetics style. Once your paper is typeset, you will receive an email with a link to choose the appropriate publishing options for your paper and our Author Services team will be in touch regarding any additional information that may be required.

After the grant of rights is completed, you will receive a link to your electronic proof via email with a request to make any corrections within 48 hours. If, when you receive your proof, you cannot meet this deadline, please inform us at rjsproduction@springernature.com immediately.

natureresearch

You will not receive your proofs until the publishing agreement has been received through our system.

Due to the importance of these deadlines, we ask that you please let us know now whether you will be difficult to contact over the next month. If this is the case, we ask you provide us with the contact information (email, phone and fax) of someone who will be able to check the proofs on your behalf, and who will be available to address any last-minute problems.

Your paper will be published online after we receive your corrections and will appear in print in the next available issue. You can find out your date of online publication by contacting the Nature Press Office (press@nature.com) after sending your e-proof corrections. Now is the time to inform your Public Relations or Press Office about your paper, as they might be interested in promoting its publication. This will allow them time to prepare an accurate and satisfactory press release. Include your manuscript tracking number (NG-AN58519R1) and the name of the journal, which they will need when they contact our Press Office.

Before your paper is published online, we shall be distributing a press release to news organizations worldwide, which may very well include details of your work. We are happy for your institution or funding agency to prepare its own press release, but it must mention the embargo date and Nature Genetics. Our Press Office may contact you closer to the time of publication, but if you or your Press Office have any enquiries in the meantime, please contact press@nature.com.

Acceptance is conditional on the data in the manuscript not being published elsewhere, or announced in the print or electronic media, until the embargo/publication date. These restrictions are not intended to deter you from presenting your data at academic meetings and conferences, but any enquiries from the media about papers not yet scheduled for publication should be referred to us.

Please note that <i>Nature Genetics</i> is a Transformative Journal (TJ). Authors may publish their research with us through the traditional subscription access route or make their paper immediately open access through payment of an article-processing charge (APC). Authors will not be required to make a final decision about access to their article until it has been accepted. Find out more about Transformative Journals

<B>Authors may need to take specific actions to achieve compliance with funder and institutional open access mandates. For submissions from January 2021, if your research is supported by a funder that requires immediate open access (e.g. according to Plan S principles) then you should select the gold OA route, and we will direct you to the compliant route where possible. For authors selecting the subscription publication route our standard licensing terms will need to be accepted, including our self-archiving policies. Those standard licensing terms will supersede any other terms that the author or any third party may assert apply to any version of the manuscript.

Please note that Nature Research offers an immediate open access option only for papers that were first submitted after 1 January, 2021.

If you have any questions about our publishing options, costs, Open Access requirements, or our legal forms, please contact ASJournals@springernature.com

If you have posted a preprint on any preprint server, please ensure that the preprint details are updated with a publication reference, including the DOI and a URL to the published version of the article on the journal website.

To assist our authors in disseminating their research to the broader community, our SharedIt initiative provides you with a unique shareable link that will allow anyone (with or without a subscription) to read the published article. Recipients of the link with a subscription will also be able to download and print the PDF.

As soon as your article is published, you will receive an automated email with your shareable link.

You can now use a single sign-on for all your accounts, view the status of all your manuscript submissions and reviews, access usage statistics for your published articles and download a record of your refereeing activity for the Nature journals.

An online order form for reprints of your paper is available at https://www.nature.com/reprints/author-reprints.html. Please let your coauthors and your institutions' public affairs office know that they are also welcome to order reprints by this method.

If you have not already done so, we invite you to upload the step-by-step protocols used in this manuscript to the Protocols Exchange, part of our on-line web resource, natureprotocols.com. If you complete the upload by the time you receive your manuscript proofs, we can insert links in your article that lead directly to the protocol details. Your protocol will be made freely available upon publication of your paper. By participating in natureprotocols.com, you are enabling researchers to more readily reproduce or adapt the methodology you use. Natureprotocols.com is fully searchable, providing your protocols and paper with increased utility and visibility. Please submit your protocol to https://protocolexchange.researchsquare.com/. After entering your nature.com username and password you will need to enter your manuscript number (NG-AN58519R1). Further information can be found at https://www.nature.com/nprot/.

Sincerely,

Wei Li, PhD
Senior Editor
Nature Genetics
New York, NY 10004, USA
www.nature.com/ng
